# Advances in automated fetal brain MRI segmentation and biometry: Insights from the FeTA 2024 challenge

**DOI:** 10.1016/j.media.2026.103941

**Published:** 2026-01-16

**Authors:** Vladyslav Zalevskyi, Thomas Sanchez, Misha Kaandorp, Margaux Roulet, Diego Fajardo-Rojas, Liu Li, Jana Hutter, Hongwei Bran Li, Matthew J. Barkovich, Hui Ji, Luca Wilhelmi, Aline Dändliker, Céline Steger, Mériam Koob, Yvan Gomez, Anton Jakovčić, Melita Klaić, Ana Adžić, Pavel Marković, Gracia Grabarić, Milan Rados, Jordina Aviles Verdera, Gregor Kasprian, Gregor Dovjak, Raphael Gaubert-Rachmühl, Maurice Aschwanden, Qi Zeng, Davood Karimi, Denis Peruzzo, Tommaso Ciceri, Giorgio Longari, Rachika E. Hamadache, Amina Bouzid, Xavier Lladó, Simone Chiarella, Gerard Martí-Juan, Miguel Ángel González Ballester, Marco Castellaro, Marco Pinamonti, Valentina Visani, Robin Cremese, Keïn Sam, Fleur Gaudfernau, Param Ahir, Mehul Parikh, Maximilian Zenk, Michael Baumgartner, Klaus Maier-Hein, Li Tianhong, Yang Hong, Zhao Longfei, Domen Preloznik, Žiga Špiclin, Jae Won Choi, Muyang Li, Jia Fu, Guotai Wang, Jingwen Jiang, Lyuyang Tong, Bo Du, Andrea Gondova, Sungmin You, Kiho Im, Abdul Qayyum, Moona Mazher, Steven A. Niederer, Andras Jakab, Roxane Licandro, Kelly Payette, Meritxell Bach Cuadra

**Affiliations:** a Department of Radiology, Lausanne University Hospital and University of Lausanne, Lausanne, Switzerland; b CIBM Center for Biomedical Imaging, Lausanne, Switzerland; c Center for MR-Research, University Children’s Hospital Zurich, University of Zurich, Zurich, Switzerland; d University of Zurich, Zurich, Switzerland; e Department of Early Life Imaging, School of Biomedical Engineering & Imaging Sciences, King’s College London, London, UK; f National Heart & Lung Institute, Imperial College London, London, UK; g Smart Imaging Lab, University Hospital Erlangen, Erlangen, Germany; h Department of Quantitative Biomedicine, University of Zurich, Zurich, Switzerland; i Department of Informatics, Technical University of Munich, Munich, Germany; j University of California San Francisco, UCSF Benioff Children’s Hospital, San Francisco, California, USA; k Neuroscience Center Zurich, University of Zurich, Zurich, Switzerland; l Department Woman-Mother-Child, CHUV, Lausanne, Switzerland; m BCNatal Fetal Medicine Research Center (Hospital Clínic and Hospital Sant Joan de Déu), Universitat de Barcelona, Barcelona, Spain; n Croatian Institute for Brain Research, School of Medicine, University of Zagreb, Zagreb, Croatia; o Department of Biomedical Engineering, School of Biomedical Engineering & Imaging Sciences, King’s College, London, UK; p Department of Biomedical Imaging and Image-Guided Therapy, Division of Neuroradiology and Musculoskeletal Radiology, Medical University of Vienna, Vienna, Austria; q Boston Children’s Hospital, Harvard Medical School, Boston, Massachusetts, USA; r Neuroimaging Unit, Scientific Institute IRCCS E. Medea, Bosisio Parini, Italy; s Department of Informatics, Systems and Communication, University of Milano Bicocca, Milan, Italy; t Research Institute of Computer Vision and Robotics (ViCOROB), Universitat de Girona, Girona, Spain; u Università di Bologna, Bologna, Italy; v BCN MedTech, Department of Engineering, Universitat Pompeu Fabra, Barcelona, Spain; w ICREA, Barcelona, Spain; x Department of Information Engineering, University of Padova, Padova, Italy; y Institut Pasteur, Université Paris Cité, CNRS UMR 3571, Decision and Bayesian Computation, Paris, France; z Inria, HeKA, PariSantéCampus, Paris, France; aa L. D. College of Engineering, Gujarat, India; ab Medical Faculty Heidelberg, Heidelberg University, Pattern Analysis and Learning Group, Department of Radiation Oncology, Heidelberg University Hospital, Heidelberg, Germany; ac German Cancer Research Center (DKFZ) Heidelberg, Division of Medical Image Computing, Heidelberg, Germany; ad Pattern Analysis and Learning Group, Department of Radiation Oncology, Heidelberg University Hospital, Heidelberg, Germany; ae Helmholtz Imaging, German Cancer Research Center (DKFZ), Heidelberg, Germany; af Faculty of Mathematics and Computer Science, Heidelberg University, Heidelberg, Germany; ag Canon Medical Systems (China) Co., Ltd, China; ah Faculty of Electrical Engineering, University of Ljubljana, Ljubljana, Slovenia; ai Department of Radiology, Seoul National University Hospital, Seoul, South Korea; aj School of Mechanical and Electrical Engineering, University of Electronic Science and Technology of China, Chengdu, China; ak School of Computer Science, Wuhan University, Wuhan, China; al Fetal Neonatal Neuroimaging and Developmental Science Center, Boston Children’s Hospital, Harvard Medical School, Boston, Massachusetts, USA; am Division of Newborn Medicine, Boston Children’s Hospital, Harvard Medical School, Boston, Massachusetts, USA; an Department of Radiology, Boston Children’s Hospital, Harvard Medical School, Boston, Massachusetts, USA; ao Hawkes Institute, Department of Computer Science, University College London, London, UK; ap University Research Priority Project Adaptive Brain Circuits in Development and Learning (AdaBD), University of Zurich, Zurich, Switzerland; aq Laboratory for Computational Neuroimaging, Athinoula A. Martinos Center for Biomedical Imaging, Massachusetts General Hospital/Harvard Medical School, Charlestown, Massachusetts, USA; ar Department of Biomedical Imaging and Image-guided Therapy, Computational Imaging Research Lab (CIR), Early Life Image Analysis Group, Medical University of Vienna, Vienna, Austria

**Keywords:** Fetal brain, MRI, Low-field, Segmentation, Topology, Biometry, Domain shift, Challenge results

## Abstract

Accurate fetal brain tissue segmentation and biometric measurement are essential for monitoring neurodevelopment and detecting abnormalities in utero. The Fetal Tissue Annotation (FeTA) Challenges have established robust multi-center benchmarks for evaluating state-of-the-art segmentation methods. This paper presents the results of the 2024 challenge edition, which introduced three key innovations.

First, we introduced a topology-aware metric based on the Euler characteristic difference (ED) to overcome the performance plateau observed with traditional metrics like Dice or Hausdorff distance (HD), as the performance of the best models in segmentation surpassed the inter-rater variability. While the best teams reached similar scores in Dice (0.81–0.82) and HD95 (2.1–2.3 mm), ED provided greater discriminative power: the winning method achieved an ED of 20.9, representing roughly a 50% improvement over the second- and third-ranked teams despite comparable Dice scores.

Second, we introduced a new 0.55T low-field MRI test set, which, when paired with high-quality super-resolution reconstruction, achieved the highest segmentation performance across all test cohorts (Dice=0.86, HD95=1.69, ED=6.26). This provides the first quantitative evidence that low-cost, low-field MRI can match or surpass high-field systems in automated fetal brain segmentation.

Third, the new biometry estimation task exposed a clear performance gap: although the best model reached a mean average percentage error (MAPE) of 7.72%, most submissions failed to outperform a simple gestational-age-based linear regression model (MAPE=9.56%), and all remained above inter-rater variability with a MAPE of 5.38%.

Finally, by analyzing the top-performing models from FeTA 2024 alongside those from previous challenge editions, we identify ensembles of 3D nnU-Net trained on both real and synthetic data with both image- and anatomy-level augmentations as the most effective approaches for fetal brain segmentation. Our quantitative analysis reveals that acquisition site, super-resolution strategy, and image quality are the primary sources of domain shift, informing recommendations to enhance the robustness and generalizability of automated fetal brain analysis methods.

## 1. Introduction

The fetal brain undergoes rapid and complex development throughout gestation, influenced by both genetic and environmental factors. Understanding this dynamic process is critical in both clinical and research domains, as neurodevelopmental disruptions are linked to congenital anomalies and long-term cognitive or physiological impairments (Griffiths et al., 2017; Ciceri et al., 2024; Van den Bergh et al., 2018). In vivo imaging biomarkers derived from ultrasonography (US) or magnetic resonance imaging (MRI) provide non-invasive and quantifiable metrics to monitor prenatal brain development. Deviations from normative patterns in these biomarkers have been associated with a range of pathologies, including corpus callosum (Marathu et al., 2024; Lamon et al., 2024) and posterior fossa malformations (Dovjak et al., 2020; Mahalingam et al., 2021), ventriculomegaly (Chen et al., 2024), and have been shown to correlate with neurodevelopmental outcomes in conditions such as congenital heart disease (Sadhwani et al., 2022), intrauterine growth restriction (Egaña-Ugrinovic et al., 2015; Meijerink et al., 2023), and preterm birth (Story et al., 2021; Hall et al., 2024).

Fetal brain MRI has emerged as an important non-invasive tool for studying neurodevelopment in utero and diagnosing congenital disorders, complementing ultrasonography (Griffiths et al., 2017; Alamo et al., 2010). Accurate and automatic segmentation of fetal brain tissues in MRI is critical for quantitative analysis and biomarker extraction, including tissue volumetry, cortical morphometry (Payette et al., 2023), and biometric measurements (She et al., 2023b). Manual segmentation, however, remains labor-intensive, error-prone, and susceptible to inter-observer variability, underscoring the necessity of reliable automated techniques.

While clinical US and 2D MRI are the standard techniques for assessing fetal development (Tilea et al., 2009), the use of super-resolution reconstruction (SRR) techniques to generate 3D fetal brain reconstructions has emerged as a powerful advancement. SRR methods fuse multiple 2D MRI slices (often motion-corrupted) into a single, enhanced 3D motion-corrected volume, significantly improving brain analysis (Gafner et al., 2020; Avisdris et al., 2021; Matthew et al., 2024). Recent studies have shown that biometric measurements derived from 3D SRR volumes correlate strongly with those from ultrasound, while offering greater rater confidence than using 2D MRI series (Lamon et al., 2024; Khawam et al., 2021; Gafner et al., 2020; Sanchez et al., 2024b; Kyriakopoulou et al., 2016; Ciceri et al., 2023).

The Fetal Tissue Annotation (FeTA) challenges, held in 2021 (Payette et al., 2023) and 2022 (Payette et al., 2024), have played a central role in advancing fetal brain MRI analysis by releasing the first publicly available dataset of real fetal MRI scans.^1^ Compared to other publicly available fetal MRI datasets (Price et al., 2019; Urru et al., 2023) FeTA offers several important advantages. Unlike the dHCP dataset (Price et al., 2019), which is based on a tailored research acquisition protocol and semi-automatically refined labels, FeTA provides annotations created entirely from scratch based on the images from standard clinical fetal MRI protocols, which rely on multi-orientation T2w stacks routinely acquired in prenatal care. This makes the dataset particularly valuable for developing clinically applicable models that perform reliably under real-world imaging conditions, as it uniquely provides both voxel-wise tissue segmentations and biometric measurements that are not available in any other fetal MRI dataset simultaneously. Compared to Price et al. (2019), Urru et al. (2023) FeTA also includes a broad spectrum of pathological cases (e.g., ventriculomegaly, spina bifida) that are essential for developing models robust to abnormal anatomy. Unlike publicly available atlases (Gholipour et al., 2017; Urru et al., 2023; Uus et al., 2023c; Bagheri et al., 2025) that aim at characterizing normal growth at the population level, FeTA offers images and annotations at the individual-subject level, thereby capturing the natural variability in brain appearance among typically developing fetuses. In addition, unlike dHCP, the FeTA dataset is multi-centric for both training and testing, encompassing data from multiple institutions, scanner manufacturers, and field strengths to promote model generalization and robustness.

Compared to the other challenge editions, FeTA 2024 retains the core brain tissue segmentation task and introduces a new clinically relevant objective: biometry extraction, alongside several other key innovations. Firstly, FeTA 2024 introduces a new low-field (LF, 0.55T) MRI testing dataset. LF MRI offers a low-cost alternative to 1.5–3T systems, making it especially valuable in resource-limited settings (Arnold et al., 2023; Marques et al., 2019). This affordability supports research in low- and middle-income countries with large pediatric populations, where access to high-field MRI is limited, hindering studies on brain development under normal and adverse conditions (Murali et al., 2023; Aviles Verdera et al., 2023). Secondly, we introduced the Euler characteristic difference as an additional ranking metric for segmentation (Taha and Hanbury, 2015). Unlike overlap- or distance-based metrics, it captures topological correctness, offering a complementary view of performance (Maier-Hein et al., 2024). This is especially relevant for downstream tasks like cortical surface extraction or morphometric analyses (e.g., sulcal/gyral folding, cortical maturation and thickness, or structural abnormality assessment) (Yehuda et al., 2023; Clouchoux et al., 2011; Mihailov et al., 2025), and accurate tissue-level segmentation remains a critical prerequisite for these downstream tasks. Robust delineation of major tissue classes enables reliable volumetric quantification, provides the anatomical boundaries required for cortical surface extraction, and supports quality control prior to more fine-grained analyses (Germanaud et al., 2012; Bouachba et al., 2025; Sanchez et al., 2025b). For instance, accurate cortical grey matter segmentation is essential for generating surfaces from which cortical thickness and folding patterns, key biomarkers of neurodevelopmental status, can be derived (Makropoulos et al., 2018). In clinical workflows, automated tissue segmentation has the potential to substantially reduce the time and variability of manual annotations, standardize biometry measurements, and facilitate longitudinal monitoring of brain growth (She et al., 2023a). **However, several barriers remain before routine adoption is possible**. These include the need for stable performance across diverse fetal neuropathologies, topologically consistent segmentations, and generalization across heterogeneous imaging conditions. Overcoming these challenges will be essential for integrating automated segmentation into prenatal diagnostic workflows, where it could inform both routine clinical practice and research into the early origins of neurodevelopmental disorders.

This paper directly addresses these challenges through the analysis of the results of the FeTA 2024 challenge. Using the BIAS reporting framework (Maier-Hein et al., 2020) we present the organization and outcomes of the challenge. Our analysis highlights the methodological advances driving state-of-the-art performance in both tissue segmentation and biometry estimation tasks and demonstrates the promise of low-field MRI for automated fetal brain analysis. We further underscore the importance of topology-aware metrics for assessing anatomical plausibility and downstream usability of segmentations. Finally, we analyze performance trends and model architectures across the last three FeTA editions and provide a detailed assessment of how domain shifts affect leading methods, offering key insights and directions for advancing automated fetal brain analysis.

## 2. Methods

### 2.1. Current state-of-the art models

Previous FeTA challenges have contributed widely to the development of state-of-the-art automated fetal brain analysis models (Uus et al., 2023d; Zalevskyi et al., 2024; Peng et al., 2023; Huang et al., 2023; Payette et al., 2024). Analyzing the methodological contributions of these works reveals that most rely on 3D Convolutional Neural Network (CNN) models like the **U-Net** (Ronneberger et al., 2015) and its self-configuring variant, **nnU-Net** (Isensee et al., 2018).

**U-Net** is an encoder-decoder style CNN architecture, in which the encoder uses repeated blocks of convolutions and max pooling to down-sample the input image, capturing high-level features while reducing spatial dimensions. The decoder (expansive path) then uses up-sampling operations to restore the image to its original size. A key feature of U-Net is its skip connections that concatenate feature maps from the encoder to the corresponding layers in the decoder. This allows the network to combine the high-level contextual information from the deeper layers with the fine-grained spatial details from the earlier layers, which is crucial for precise segmentation.

A prominent example of U-Net applications for fetal brain segmentation is the **BOUNTI** model (Uus et al., 2023d), which uses a combination of a classical U-Net and an Attention U-Net (Oktay et al., 2018), which integrates attention gates to filter and weigh feature maps before concatenation in the skip-connections. BOUNTI was trained on a large and diverse multi-centric dataset of 360 T2w images, with training annotations refined through a semi-supervised approach. Another notable model using U-Net architecture is **FetalSynthSeg** (Zalevskyi et al., 2024), which focuses on maximizing data diversity rather than increasing dataset size. It incorporated advanced data augmentation strategies based on SynthSeg (Billot et al., 2023) to compensate for the limited available training data, showing great out-of-domain generalization performance in fetal imaging.

The top three models of FeTA 2022 all adopted **nnU-Net** (Isensee et al., 2018), an auto-configuring segmentation framework that automatically adjusts U-Net model’s architecture depth, convolution kernel sizes, patch sizes, normalization layers, learning rates, and data augmentation strategies based on dataset properties. These teams also employed ensembling and post-processing techniques, including denoising autoencoders (**DAEs**) to remove spurious segmentation artifacts. DAE is a type of neural network that learns to reconstruct a clean output from a corrupted, noisy input, and by training it to denoise segmentation masks, it can refine and improve the final output, removing spurious artifacts and inconsistencies (Gondara, 2016).

While some teams in FeTA 2022 have experimented with Transformer-based models, such as **Swin Transformer** backbones (Hatamizadeh et al., 2022), which process images as sequences of patches and model long-range dependencies via multi-head self-attention, their performance has generally lagged behind 3D CNNs in this task. This can be attributed to the fundamental differences in how these two architectures operate. CNNs, with their inductive biases like weight sharing and local receptive fields, are inherently effective at capturing local spatial hierarchies and fine-grained details, which are critical for delineating precise tissue boundaries in medical images (Ronneberger et al., 2015). Transformers, originally developed for natural language processing (Vaswani, 2017), use self-attention mechanisms to model global dependencies and long-range relationships. However, they typically require larger datasets to learn meaningful representations and are more computationally expensive. In data-limited scenarios like fetal brain analysis, CNNs often outperform Transformers by leveraging their strong local feature extraction capabilities, as shown in the previous FeTA editions (Payette et al., 2024).

Across FeTA submissions and related works, **data augmentation plays a central role**. For instance, BOUNTI relied on standard MONAI transformations (bias field simulation, affine rotation/scaling, Gaussian noise addition, and Gaussian blurring), which sufficed given its large, diverse dataset. By contrast, FetalSynthSeg was trained on fewer subjects, and thus employed a more complex data augmentation pipeline based on the domain randomization approach of **SynthSeg** (Billot et al., 2023). SynthSeg generates synthetic images from input segmentations with randomized intensities and contrast, resolution, and noise properties, enabling models to learn contrast- and resolution-invariant features. The FeTA 2022 winning team used **style augmentation** via a pretrained on ImageNet neural style transfer model (Jackson et al., 2019) to alter image appearance while preserving anatomical structure, along with **Global Intensity Non-linearity (GIN)** augmentation (Ouyang et al., 2022), which applies non-linear intensity mappings to simulate images with diverse appearances through a family of randomly-weighted shallow networks.

In **fetal brain biometry estimation**, current methods can be broadly grouped into three categories: **tissue segmentation-based approaches** (She et al., 2023b), which first identify relevant brain tissues or structures and then derive measurements such as biparietal diameter or head circumference from the resulting masks; **registration-based approaches** (Matthew et al., 2024), which align an annotated atlas to the target image and transfer biometric landmarks for measurement; and **direct regression or localization approaches** (Luis et al., 2025), which estimate biometric values or landmark coordinates directly from the image without intermediate segmentation or registration. Across all categories, 3D U-Net variants remain the dominant choice, used both for tissue segmentation as a preprocessing step and for keypoint detection, due to their strong performance-efficiency trade-off in data-limited scenarios, particularly as the task of automated fetal brain MRI biometry estimation is still in its early stages of development.

### 2.2. Challenge organization

#### Context.

The FeTA 2024 challenge was held as a thematic event within the Perinatal, Preterm, and Pediatric Image Analysis (PIPPI) workshop,^2^ part of the Medical Image Computing and Computer-Assisted Intervention (MICCAI) 2024 conference. The challenge was run through a custom platform, available at https://fetachallenge.github.io/, which provided participants with all the necessary information on the organization, time frame, and submission instructions.

#### Data, participation and submission.

Challenge participation required submission of **fully automated** segmentation and/or biometry algorithms. A training set of 3D super-resolution fetal brain MRI from two institutions was provided; no validation set was released, and test data remained private for evaluation. Participants could use publicly available external datasets and pre-trained models, provided these were public and fully documented in the algorithm description, as well as use both 2D and 3D models.

Participants submitted their algorithms as Docker containers with a command-line interface for test data evaluation.^3^ Any programming language was allowed, provided the input/output followed the evaluation utility specifications. Each team was allowed one submission, except in cases of technical errors (e.g., Docker issues), which could be corrected upon notification by the organizers. Evaluation on test data was performed by the organizers using publicly available code.^4^ To promote transparency and reproducibility, FeTA 2024 encouraged participants to share their code publicly. A Docker Hub page (https://hub.docker.com/repositories/fetachallenge2024) was created to host containers from teams who agreed to release their Docker images.

#### Timetable, rewards and results paper.

The challenge followed a predefined schedule: training data was released on May 21, 2024; registration opened after challenge acceptance. The Docker submission deadline was extended to August 4, 2024, and algorithm descriptions were due by August 12. On August 23, the top five teams were invited to prepare 2-minute pitch presentations for the challenge day and, along with all participants, were invited to present posters at the dedicated conference session. The challenge took place in person on October 6, 2024, during MICCAI. Results were announced live and later published on the challenge website, along with top teams’ presentations (with their consent). The top three teams in each task received certificates and small gifts, including a 3D-printed fetal brain keychain for in-person attendees. The highest-ranking team in each task also received a box of artisanal Swiss-made cookies. Organizers could participate but were not eligible for awards.

All teams with valid submissions and interest in the publication were included in this results paper, with up to three members per team listed as co-authors. Teams were free to publish their algorithms and results independently after the challenge, without embargo, provided they cited both the data publication (Payette et al., 2021) and this summary paper.

#### Data usage terms and conflicts of interest.

The training data from the University Children’s Hospital Zürich (**Kispi**) and General Hospital Vienna/Medical University of Vienna were provided with specific licensing conditions. Kispi data, hosted on the Synapse platform,^5^ **is for non-commercial use only**. **Vienna data** is governed by a custom Data Transfer Agreement, **allowing use for challenge purposes only**. Participants could modify the data, including generating synthetic data through augmentation, as long as modifications were documented and synthetic data could be provided to the organizers upon request. None of the organizers participated in this year’s challenge or have conflicts of interest to disclose. The challenge awards were funded by the institutional budget (Kispi), and none of the participants were involved in funding. Only organizers at Kispi had full access to the testing dataset, as they managed data transfer agreements with all providers.

### 2.3. Challenge tasks

**Fig. 1.**
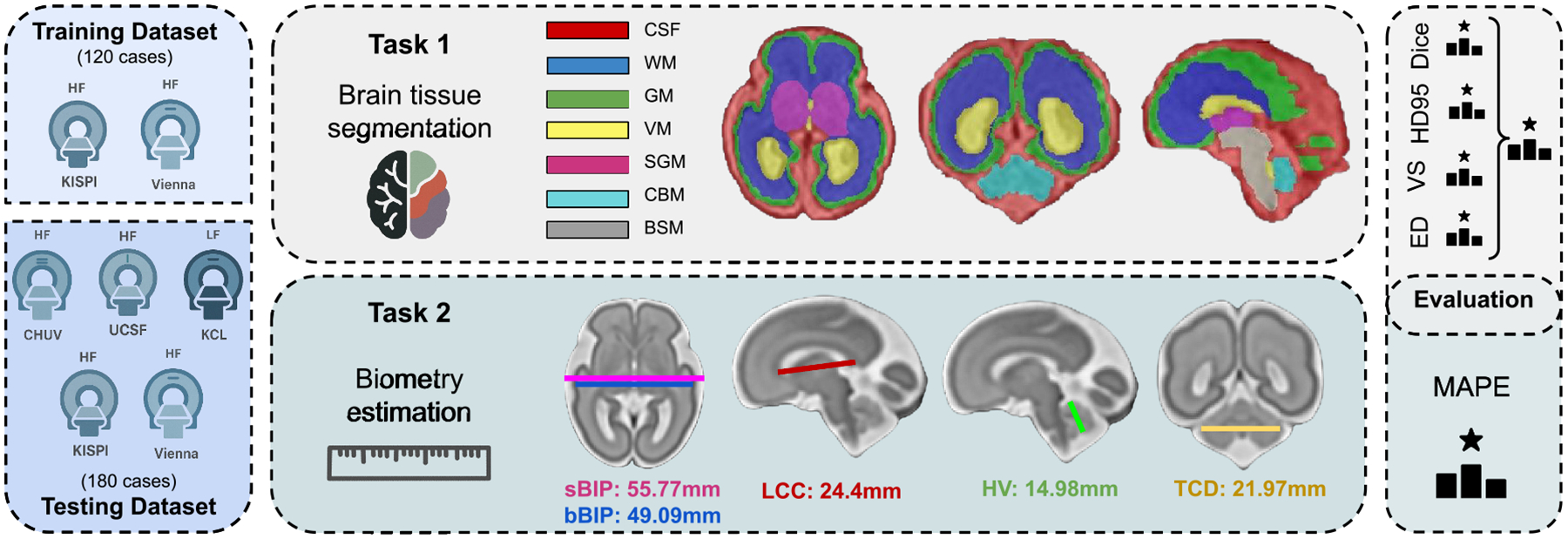
FeTA 2024 challenge organization structure. The challenge provides a diverse, multi-center dataset comprising 300 super-resolution fetal brain MRIs. Participants could take part in either Task 1, which involves segmentation of fetal brain tissues into seven classes, or Task 2, which focuses on estimating five biometric measurements. Finally, Task 1 submissions were ranked based on the average rank across four evaluation metrics, while Task 2 was ranked using the mean absolute percentage error (MAPE).

The FeTA challenge presents two primary tasks (see Fig. 1). Participants could choose to compete in either or both tasks.

#### Task 1. Fetal brain tissue segmentation.

This task aims to develop algorithms that automatically delineate different tissues in SRR fetal brain MRI. The 3D semantic segmentation involves classifying each voxel into one of seven predefined classes: Background, External CSF, Grey Matter (GM), White Matter (WM), Ventricles including cavum (VM), Cerebellum (CBM), Deep Grey Matter (SGM), and Brainstem (BSM). Reference annotation procedures and inter-rater variability analyses for all datasets (except the new LF set) are detailed in Payette et al. (2021) and Payette et al. (2024). The LF dataset followed the same annotation protocol, with seven annotators (AJ, CS, RG, VZ, YG, MA, MR) each segmenting a specific label map. These were merged into a single reference annotation, reviewed, and corrected by two fetal MRI experts (KP, AJ).

#### Task 2. Biometric measurements prediction.

The goal of this task is to develop algorithms that automatically and accurately estimate key fetal brain biometry from MRI. The selected measurements-length of the corpus callosum (LCC), height of the vermis (HV), brain biparietal diameter (bBIP), skull biparietal diameter (sBIP), and transverse cerebellar diameter (TCD)-were chosen to **minimize annotation burden** while providing **complementary anatomical and diagnostic value**. Four raters contributed: YG (5 years’ fetal MRI experience), MKo (16 years), and junior raters RG and MA (reviewed by AJ, 12 years).^6^ Not all measurements were available for all cases: in the test set, 15 cases lacked LCC, one lacked HV, and one lacked TCD due to annotator uncertainty. In the training set, 102 of 120 cases had complete annotations-10 Kispi cases were excluded for poor quality; 5 Kispi and 3 Vienna cases had partial annotations. While the main goal was to predict biometry values, the training set also included 3D **landmark annotations**-single-voxel labels marking anatomical structures used to derive each measurement. Clinicians identified these landmarks during annotation, and the actual biometry values were computed via organizer-provided scripts. Both the landmarks and scripts were shared, allowing participants to either regress biometry directly or predict landmarks, followed by automated biometry measurement.

### 2.4. Challenge data sets

Subject selection aimed to ensure a representative cohort spanning 18–35 weeks of gestation, including both neurotypical and pathological cases (e.g., spina bifida, ventriculomegaly, corpus callosum malformations) to reflect clinical practice. UCSF and CHUV data were acquired during clinical fetal MRI scans following ultrasound referral, performed by trained medical staff. Data from KCL, Kispi, and Vienna were collected using research protocols. All cohorts had approval by the local ethics committee for use in the challenge after anonymization.^7^

Each case included a 3D fetal brain MRI reconstruction, manual brain tissue segmentation, and biometry annotations. Metadata included gestational age (GA) and a binary label indicating neurotypical or pathological status. To preserve anonymity, gender was excluded and GA was randomly offset by ±3 days.

**Fig. 2.**
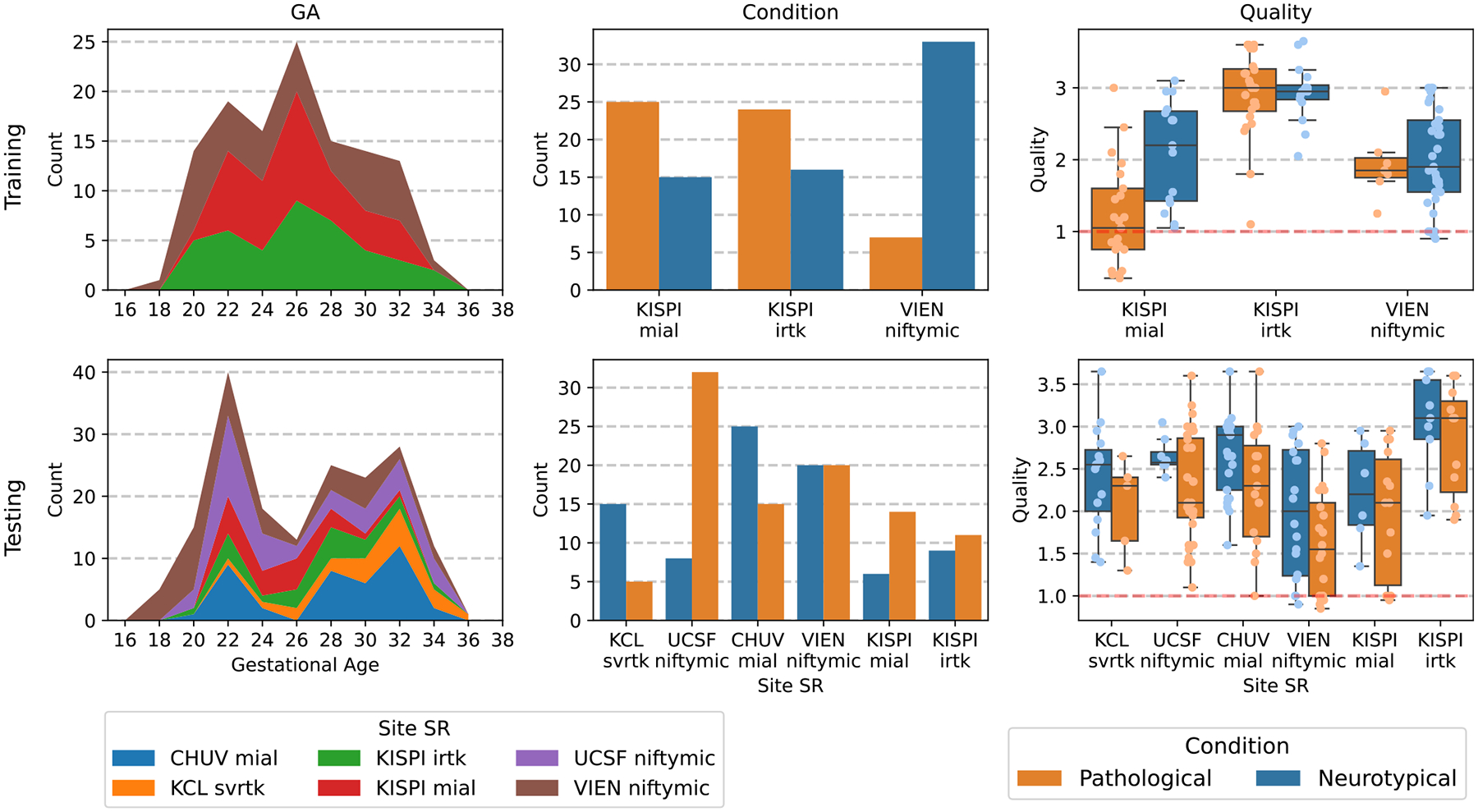
FeTA 2024 data distribution by GA (weeks), condition, and image quality (0 = lowest, 4 = highest; 1 = minimum acceptable), stratified by Site and SRR method for training (top) and testing (bottom) sets. In the image quality plots, the red dotted line marks the threshold score (1.0); images with a score below this value are classified as poor quality.

The challenge dataset comprises of 120 training and 180 test cases. The test set was split into *in-domain* (from the same institutions and protocols as training data) and *out-of-domain* cases. To ensure balance, both subsets were similar in size to the training set. Demographic characteristics, including GA and pathology distribution, were matched across training and testing cohorts (see Fig. 2). For this year’s challenge, manual quality control was performed on all training and testing cases following the protocol by Sanchez et al. (2024a), Bach Cuadra et al. (2025), ensuring comparable data quality between training and testing sets (Fig. 2).

The FeTA 2024 training and testing datasets are identical to those used in FeTA 2022, with two additions: a new low-field out-of-domain test set from King’s College London (KCL) and manual biometry annotations.

All cases were acquired using T2-weighted single-shot fast spin-echo sequences,^8^ the standard for structural fetal MRI due to their high signal-to-noise ratio and reduced sensitivity to fetal motion. To further mitigate motion artifacts, multiple stacks were acquired in various orientations (axial, sagittal, coronal, and off-plane). Manual selection of 2D stacks was done at each site and then combined into a single high-resolution, isotropic 3D image via super-resolution reconstruction. The resulting 3D volumes were zero-padded to 256×256×256 and reoriented to a standard radiological plane. A summary of acquisition parameters, demographic characteristics, and reconstruction methods in all sites is provided in Table 1. Additional details about the new KCL dataset are provided below.

To avoid redundancy with our previous work (Payette et al., 2024), we have provided detailed acquisition protocols for the T2w SSFSE sequences, including scanner-specific settings and motion management procedures in Appendix A10 of the supplementary material.

Data from **KCL** was collected using a 0.55T low-field MRI scanner (Siemens MAGNETOM Free.Max) with a HASTE sequence as part of a prospective single-center study and fully anonymized following local procedures (Ethics Committee Dulwich 19 LO 0852). The acquired stacks had a resolution of 1.5mm × 1.5mm × 4.5mm, which were then reconstructed into a high-resolution volume of 0.8mm × 0.8mm × 0.8mm using SVRTK (Uus et al., 2020). Key acquisition parameters include a flip angle of 180°, a field-of-view of 450 × 450mm^2^, and a base resolution of 304×304 pixels, yielding a voxel size of 1.5 × 1.5 × 4.5mm^3^. The acquisition time ranged from 64 to 122 seconds. Data collection took place at St Thomas’ Hospital in London, United Kingdom, without the use of maternal or fetal sedation. All acquisitions were performed using the contour L coil and the integrated spine coil while the mother was in a supine position. This dataset is used only in the testing set.

### 2.5. Evaluation metrics

We provide a short recall of the ranking metrics. The detailed mathematical formulation is available in supplementary materials A1.

#### Task 1. Segmentation.

Segmentation performance is evaluated using a set of complementary metrics that capture different aspects of agreement between predicted and reference annotations: voxel-wise spatial overlap (Dice), volumetric agreement (VS), boundary accuracy (HD95), and topological plausibility (ED). Together, these metrics provide a more comprehensive assessment than any single measure alone, since strong overlap can still mask errors in shape, boundary precision, or anatomical topology.

- **Dice Similarity Coefficient** (**Dice**; ↑)^9^: measures voxel-wise correspondence between the predicted and ground truth (GT) segmentations (Zou et al., 2004).
- **Volume Similarity (VS**; ↑**):** measures the similarity of the volumes between the predicted and GT segmentations (Zou et al., 2004).
- **Hausdorff Distance (HD95**; ↓**):** quantifies the distance between contours of the predicted and GT segmentations with robustness to outliers. It measures the maximum distance between the surfaces of two segmentations. Given two sets of points *A* and *B* representing the predicted and reference segmentation boundaries, the directed Hausdorff distance is defined as Beauchemin et al. (1998):
$$h\left(A,B\right)=\underset{a\in A}{\text{max}}\;\underset{b\in B}{\text{min}}\|a-b{\|}_{2}.$$
  The symmetric Hausdorff distance is:
$$H\left(A,B\right)=\text{max}\left\{h\left(A,B\right),h\left(B,A\right)\right\}.$$
  Since *H*(*A, B*) is highly sensitive to outliers, we use the 95th percentile of all surface distances (*HD95*) instead of the maximum.
- **Euler Characteristics Difference (ED;** ↓**)**: evaluates the topological similarity between the predicted and GT segmentations (Li et al., 2025).

ED is based on the Euler characteristic (EC):

$$EC={\text{BN}}_{0}-{\text{BN}}_{1}+{\text{BN}}_{2}$$

where Betti Number BN_0_ represents the number of connected components (i.e., regions), BN_1_ represents the number of loops or holes and BN_2_ represents the number of voids or cavities. The ED difference is then computed as |*EC*_*pred*_ – *EC*_*GT*_*|*. Smaller differences indicate better topological alignment. The Betti number values of GT are: for all brain tissue labels, BN_1_ = 0 and BN_2_ = 0. For the eCSF, WM, ventricles, cerebellum, dGM, and brainstem, BN_0_ = 1, while for GM, BN_0_ = 2. In the supplementary materials (Appendix A1), we provide additional anatomical justification for the chosen ground-truth Betti numbers. For the predicted segmentations, Betti numbers were computed using a topological data analysis framework based on persistent homology applied to a cubical complex constructed from the 3D voxel grid. Each binary segmentation mask was treated as a scalar field and analyzed to quantify stable topological features. The implementation of the Euler characteristic difference metric follows the publicly available code at: https://github.com/smilell/Topology-Evaluation/tree/main.

#### Task 2: Biometry estimation.

The primary metric for evaluating biometry estimation algorithms is **mean average percentage error (MAPE;** ↓**)**, which quantifies the error in the estimated biometric measurements relative to the actual measurements:

$$\text{MAPE}=\frac{1}{N}\overset{N}{\underset{i=1}{\sum }}\frac{\left|y_{i}-{\overset{ˆ}{y}}_{i}\right|}{y_{i}}\times 100,$$

where *y*_*i*_ and ${\overset{ˆ}{y}}_{i}$ are GT and predicted measurements respectively, and *N* is the total number of measurements. This metric accounts for variable sizes of the target structures and is used to assess the accuracy of the estimated biometric measurements.

### 2.6. Ranking

Submissions are ranked based on metrics computed for each brain tissue label (or biometric measurement) in the predicted maps of the fetal brain volumes. For segmentation, the final rank is the average of all 4 metrics: Dice, HD95, VS, and ED. For biometry, the final rank is based on MAPE. For metrics where higher values are better (Dice, VS), the algorithm with the highest value ranks best. For metrics where lower values are better (like HD95 and ED for segmentation and MAPE for biometry tasks), the algorithm with the lowest value ranks best. The individual label rankings are summed, and the algorithm with the highest combined rank is considered the best.

In cases of missing results (e.g., if an algorithm fails to detect a label or if the entire label map is empty), the worst possible values will be assigned to the algorithm. For example, if a label is missing in the label map, it will receive a Dice and VS of 0. For HD95, EC, and MAPE, the missing values are set to double the maximum value of other algorithms for that sub-ranking. This ensures that algorithms with missing results are ranked last for that specific task/brain tissue.

#### 2.6.1. Biometry baselines

In the ranking of Task 2, two additional baseline models representing lower and upper performance limits were incorporated as separate submissions. These entries, intended solely for benchmarking purposes, were not considered in the formal determination of the challenge competition ranking.

##### Lower bound: Gestational age regression model.

This *model*, referred to as **[GA]** in the result’s Table 5, is a simple univariate linear regression baseline. For each biometric measurement *y*, the model predicts its value $\overset{ˆ}{y}$ using the gestational age (GA) as the sole explanatory variable, mathematically:

$$\overset{ˆ}{y}=\beta_{0}+\beta_{1}\cdot \text{GA},$$

where *β*_0_ is the intercept and *β*_1_ is the regression coefficient learned from the training data. This baseline does not rely on the image and aims at quantifying how strongly the GA can account for the size of a given structure.

##### Upper bound: Inter-rater variability.

The upper bound is set by averaging inter-rater variability, further denoted as **[inter-rater]**. This reflects the best-expected accuracy, accounting for measurement errors and uncertainties between manual raters. For each biometric measurement in the test dataset, annotations from two independent observers are used by comparing one observer’s measurement to the other’s, with the result averaged across all test cases.

### 2.7. Statistical analysis

The non-parametric **Wilcoxon signed-rank** test was used to assess performance differences between algorithms, as the Shapiro-Wilk test indicated non-normal distribution. To evaluate performance differences across subsets (e.g., neurotypical vs. pathological cohorts), we applied the **Mann-Whitney** *U* **test** (Wilcoxon rank-sum test). For all tests, statistical significance was set at *p* < .05. For multiple comparisons, such as between sites or labels, we applied **Bonferroni correction**.

### 2.8. Further analysis

FeTA 2024, as the third edition of the challenge, provides an opportunity to assess progress and unsolved challenges. We report two additional analyses: **(i)** the evolution of top-performing segmentation models over the last three editions, and **(ii)** the impact of different domain shift sources on model performance.

#### 2.8.1. Insights from three years of competition: Progress or plateau?

To assess progress in fetal brain tissue segmentation, we analyze the evolution of top-performing algorithms over time. Specifically, we compare the performance of the highest-ranked teams from the FeTA challenges in 2021, 2022, and 2024, evaluating segmentation accuracy across the dataset splits available in each respective year.

To extend the longitudinal comparison, we perform a retrospective evaluation of the 2022 winning method on the KCL dataset, first introduced as a test set in 2024. This is enabled by the 2022 winning team’s release of their Docker container,^10^ allowing us to assess the generalization of a previously state-of-the-art solution to new, unseen data, and to identify both progress and persistent limitations.

#### 2.8.2. Quantifying domain shifts

Domain shifts remain a key obstacle in fetal brain MRI analysis, often undermining model generalizability. These shifts arise from variations in subject demographics, imaging protocols, scanner types, and reconstruction methods (Dockès et al., 2021). In fetal imaging, GA notably affects brain morphology and contrast, while pathologies such as ventriculomegaly, for example, can significantly alter anatomical structure. Beyond biological and acquisition-related variability, low contrast or motion artifacts can degrade reconstruction quality, adversely affecting segmentation and biometry.

##### Is image quality a domain in itself?

To assess whether image quality impacts model generalization, we manually rated the quality of all 180 test volumes using the protocol from Sanchez et al. (2024a) and explored the interaction of data quality with the performance of the submitted algorithms across the test data.

##### Comparing the impact of domain shift factors.

**Fig. 3.**
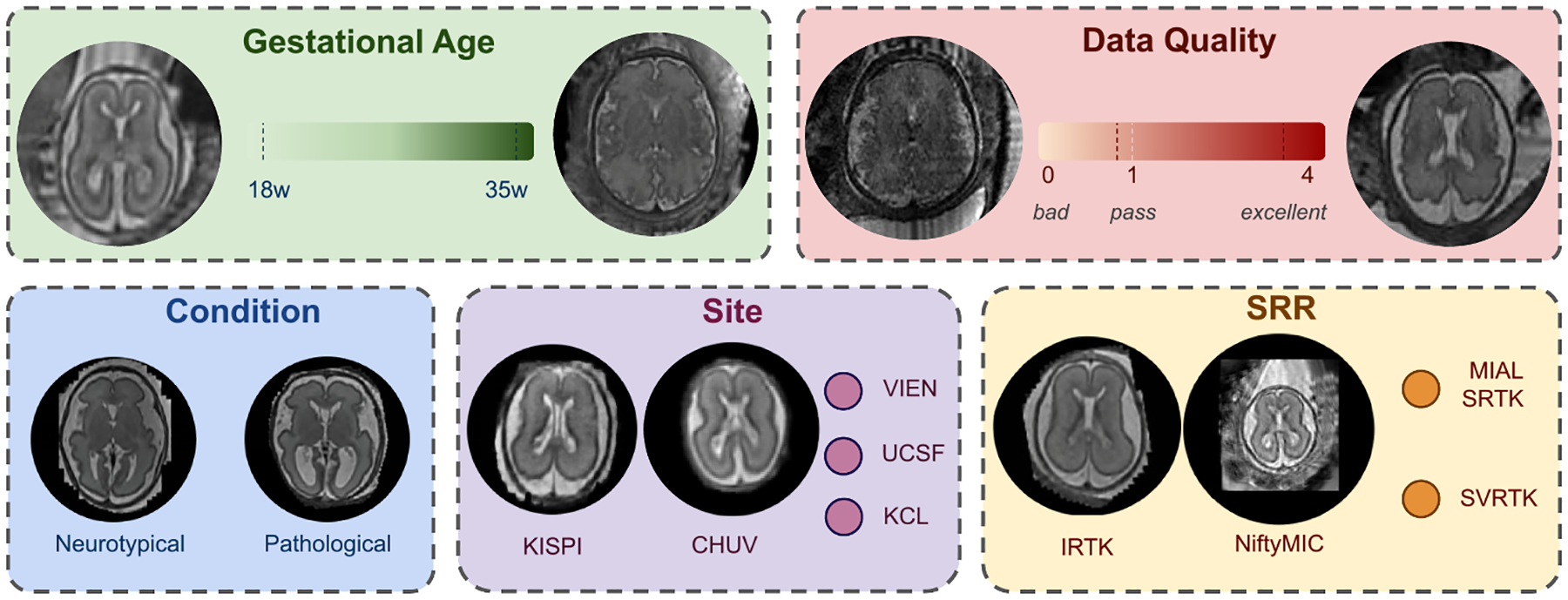
Illustration of the sources of domain shifts in fetal brain MRI datasets of FeTA 2024. Demonstrated across gestational age (18 vs. 35 weeks), data quality (0.9 vs. 3.64), clinical condition (neurotypical vs. pathological), acquisition site (KISPI vs. CHUV), and SRR methods (IRTK vs. NiftyMIC). In each comparison, only the indicated domain is varied, while all other domains remain constant. Additional domains within each source, not shown here, are represented by circles.

To assess how domain shifts influence segmentation performance, we examined four key sources of variability: image quality, GA, condition (neurotypical or pathological), as well as site and SRR together, as our dataset mostly features unique site and SRR combinations. These factors are summarized in Fig. 3. To evaluate the influence of domain shift factors on segmentation performance, we trained a random forest regressor for each metric of interest (Dice, HD95, Volume Similarity, Euler Difference), using four dataset-level variables as input features. Target values were defined as the average metric scores across the top 3 teams. To estimate feature importance, we applied SHapley Additive exPlanations (SHAP) (Lundberg and Lee, 2017), which quantify the contribution of each feature by computing its average marginal effect across all possible feature combinations. This approach provides a unified and interpretable measure of how each factor affects performance.

## 3. Results

### 3.1. Participation statistics

In total, we received 176 access requests for the KISPI training cohort hosted on Synapse during the challenge active period (May-July 2024). However, not all of these requests were related to the FeTA challenge, as the dataset is also available for broader research purposes. For the Vienna dataset, 53 data access applications were submitted, but only 30 applicants completed the data transfer agreement process and successfully received the data.

For the segmentation task, we received 16 valid submissions, all evaluated on the full test set. One team declined participation in this paper and was excluded from the analysis; results from the remaining 15 teams are presented. For the biometry task, 7 teams submitted results. One team (falcons) failed to generate valid outputs for all test cases and was penalized accordingly, as described in the Section 2.6. Notably, all biometry participants also submitted segmentation entries, leveraging segmentation outputs either as a preprocessing step or direct input for biometry estimation. A summary of each submission’s model architecture, data augmentation, ensembling strategies, and other key design choices is provided in Tables 2 and 3. Additional details, including a complete specification form with hyperparameter descriptions and values, as well as an extended overview of the employed methodology, are available in Appendix A2 of the supplementary material. We also note that 14 of the 16 participating teams agreed to share their work, and their Docker images, containing code, model weights, and configuration files, have been published on the challenge DockerHub page to ensure full reproducibility.

### 3.2. Submitted algorithms

#### 3.2.1. Segmentation models

Among the 16 submissions, the most common architectures were **nnU-Net** (Isensee et al., 2018) (9 teams) and **U-Net** (Ronneberger et al., 2015; Çiçek et al., 2016) (6 teams), often used as baselines. Many teams enhanced these models with **attention mechanisms** (Vaswani, 2017; Oktay et al., 2018), **residual connections** (He et al., 2016), or **ensembling**. Others explored alternatives such as **Swin Transformers** (Liu et al., 2021), custom U-Net variants, or hybrid CNN-Transformer designs. Most models were developed in **PyTorch** (12 teams), with parameter counts ranging from 5M to 140M (median: 31M, mean: 44.8M).

Use of **external data** was limited to 5 teams, primarily leveraging dHCP data (Hughes et al., 2016; Edwards et al., 2022), fetal brain atlases (Gholipour et al., 2017; Uus et al., 2023a), or foundation models pretrained on large-scale image datasets (Roy et al., 2023).

#### 3.2.2. Biometry models

All biometry models leveraged segmentation outputs, either as pre-processing, auxiliary, or core input. Two teams employed nnU-Net or U-Net variants for direct regression, while others used custom CNNs (1/7) or more complex architectures integrating attention mechanisms or hybrid designs (4/7). Prediction strategies varied across teams: two teams directly regressed biometry values; three teams predicted 3D landmark coordinates; and two teams generated 3D landmark heatmaps. In the latter two approaches, biometry values were subsequently computed using scripts provided by the organizers. Most teams used 3D models (6/7), implemented primarily in **PyTorch** (6/7), with one using TensorFlow. Three teams leveraged **external data**, such as dHCP and fetal brain atlases, or employed foundation models pre-trained on large-scale datasets.

#### 3.2.3. Common data and model augmentation strategies across tasks

Participants adopted a variety of approaches, with the majority utilizing 3D architectures-14 out of 16 for the segmentation task and 6 out of 7 for the biometry task. Across both tasks, two strategies were commonly used: data augmentation and model ensembling.

**Data augmentation** was universally applied, with all segmentation (16 teams) and biometry (7 teams) models using it. Standard transformations like flipping, rotation, scaling, and intensity shifts were common, while advanced methods, such as SynthSeg (Billot et al., 2023) or global intensity non-linear augmentations (GIN) (Ouyang et al., 2022), were used by 3 teams. Some teams also simulated domain-specific artifacts, including fetal motion and bias field.

**Ensembling** was a key approach in segmentation, used by 14 out of 16 teams. This included combining models trained on different cross-validation splits (4 teams) or using varied architectures, training setups, data orientations, or augmentation schemes (8 teams). Some also integrated pre- or post-processing models, like denoising autoencoders or skull-stripping (2 teams). Ensembling was less common in the biometry task, with only 2 out of 7 teams employing it, as most models built biometry predictions in a single pipeline on top of segmentation outputs.

### 3.3. FeTA 2024 results

#### 3.3.1. Brain tissue segmentation ranking

##### Segmentation performance overview.

**Fig. 4.**
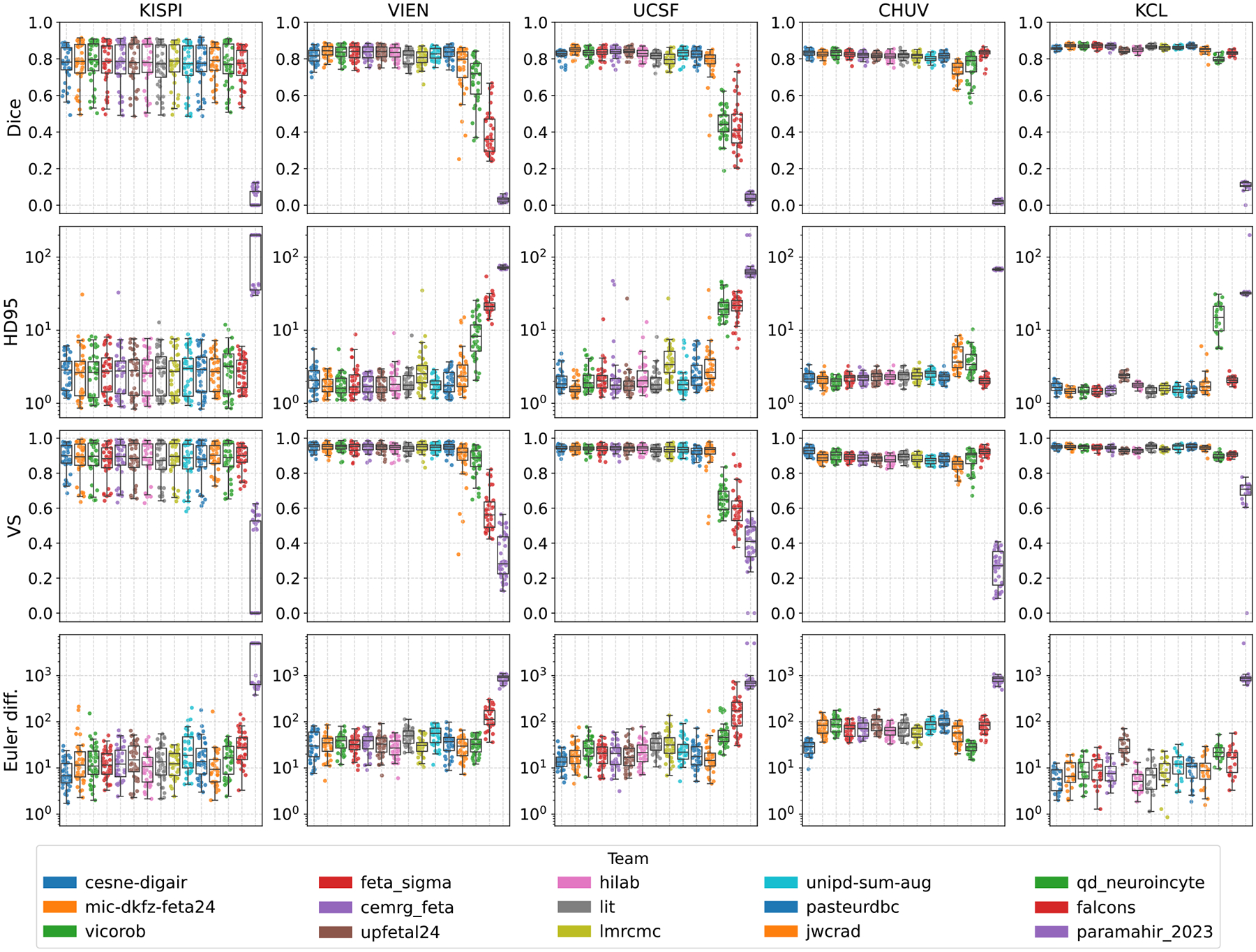
Segmentation performance by site and evaluation metric. In each subplot, teams are ranked from left to right based on their average performance across all labels for the given metric (best to worst). Team colors are consistent across plots and correspond to the legend.

Fig. 4 highlights performance across sites and metrics, revealing a general **performance plateau** among top methods. For most teams, average Dice scores stabilized around **0.8–0.82**, HD95 around **2.8–2.1**, and VS around **0.9–0.92**, while the ED showed wider variability (ranging from **20** to **40**), highlighting its sensitivity to topological inaccuracies not captured by other metrics.

The final rankings, summarized in Table 4, show a clear trend favoring CNN-based architectures. The top three teams (cesne-digair, mic-dkfz-feta24, vicorob) all employed variations of the 3D nnU-Net framework. In contrast, approaches using Transformer-based backbones or 2D models have demonstrated lower overall performance. For instance, some of these models (qd_neuroincyte,falcons) experienced significant performance drops on specific sites, with Dice scores falling to 0.38–0.44 on UCSF or VIEN data, compared to 0.76–0.83 on other sites.

Notably, post-processing played a critical role in the final ranking. The winning team, cesne-digair, incorporated a denoising autoencoder into their ensemble pipeline, which resulted in a 50% improvement in the ED score compared to the second-best submission, underscoring the impact of topology-focused refinements.

##### Site-specific trends.

Despite being introduced in this edition as a new low-field, out-of-domain dataset, KCL showed the best segmentation performance. In contrast, KISPI yielded the lowest performance, even though it was part of the previous editions’ training and testing data. Across metrics, UCSF and KISPI displayed higher interquartile ranges, particularly for Dice, HD95, and VS, reflecting greater variability across methods.

##### Label-specific trends.

SGM, GM, and BS were consistently the most challenging labels to segment across all teams, as shown by lower performance metrics in Supplementary Materials A4. Among the top three models, Dice scores dropped from an average (across all labels) of 0.82 to 0.80 for SGM, 0.79 for BS, and 0.74 for GM. HD95 increased from 2.24 to 3.6 for BS and 3.0 for SGM, while VS declined from 0.92 to 0.86 for SGM and 0.88 for BS. GM also showed a marked increase in ED, from 33.14 to 137, reflecting a significant loss in topological accuracy.

##### Ranking summary.

**Fig. 5.**
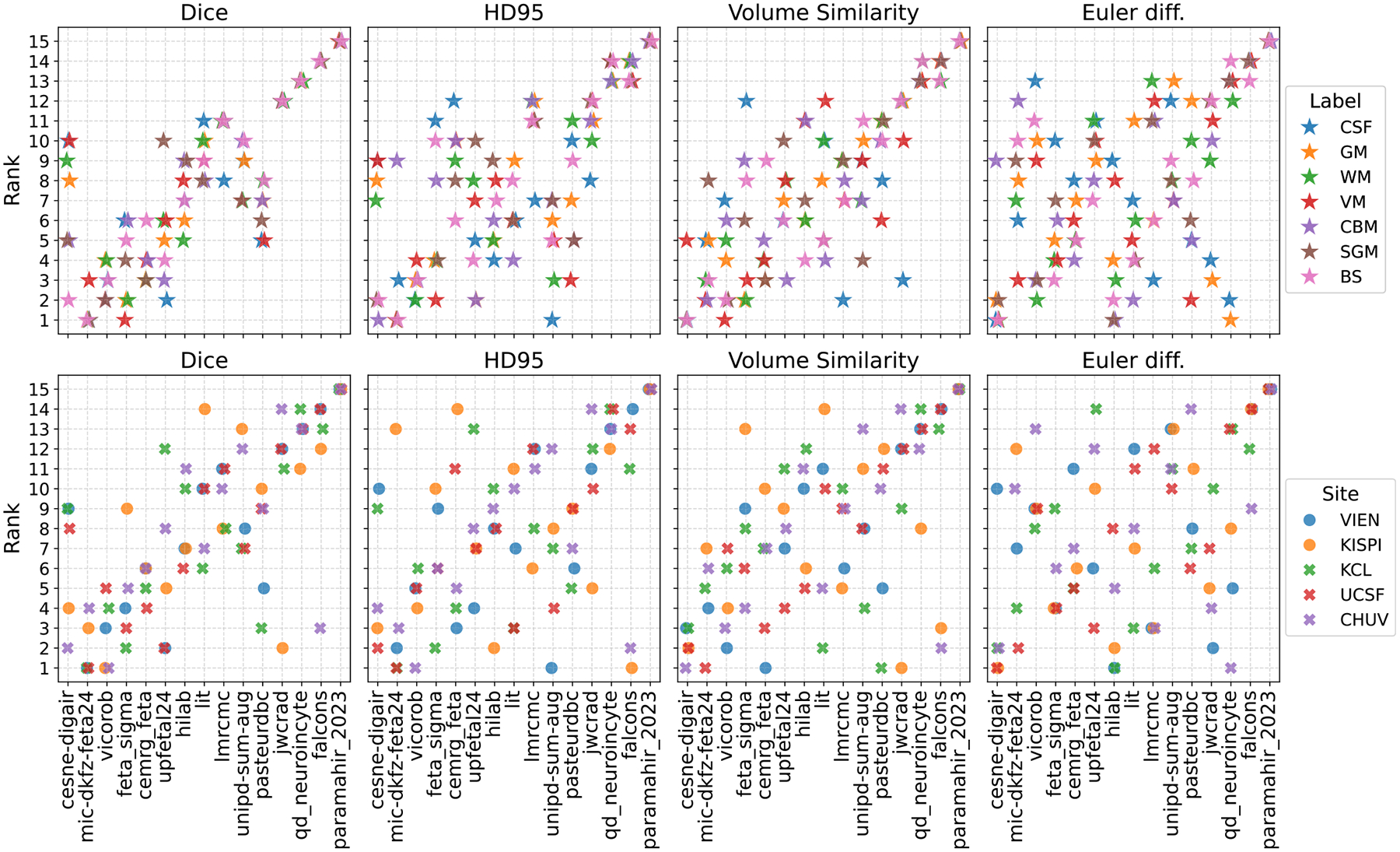
Detailed submissions rankings. Top: Across labels. Bottom: Across sites (circles indicated in-domain splits and crosses out-of-domain splits). Teams are sorted in each subplot by their final ranking in the segmentation task, from the best to the worst.

Table 4 presents the aggregated average metrics calculated across all seven tissue classes and all test subjects, along with the rankings for each team for each metric individually, as well as the final ranking.^11^ Qualitative examples of the segmentations are provided in Supplementary Appendix A9. Notable rank discrepancies across metrics highlight their complementary nature. Fig. 5 provides a more granular view, showing single-metric rankings across different sites and tissue labels. Dice score rankings remained relatively consistent across submissions and anatomical regions, while ED rankings showed greater variability, both across tissues and sites, reinforcing the importance of using multiple metrics to capture distinct aspects of segmentation quality.

**Fig. 6.**
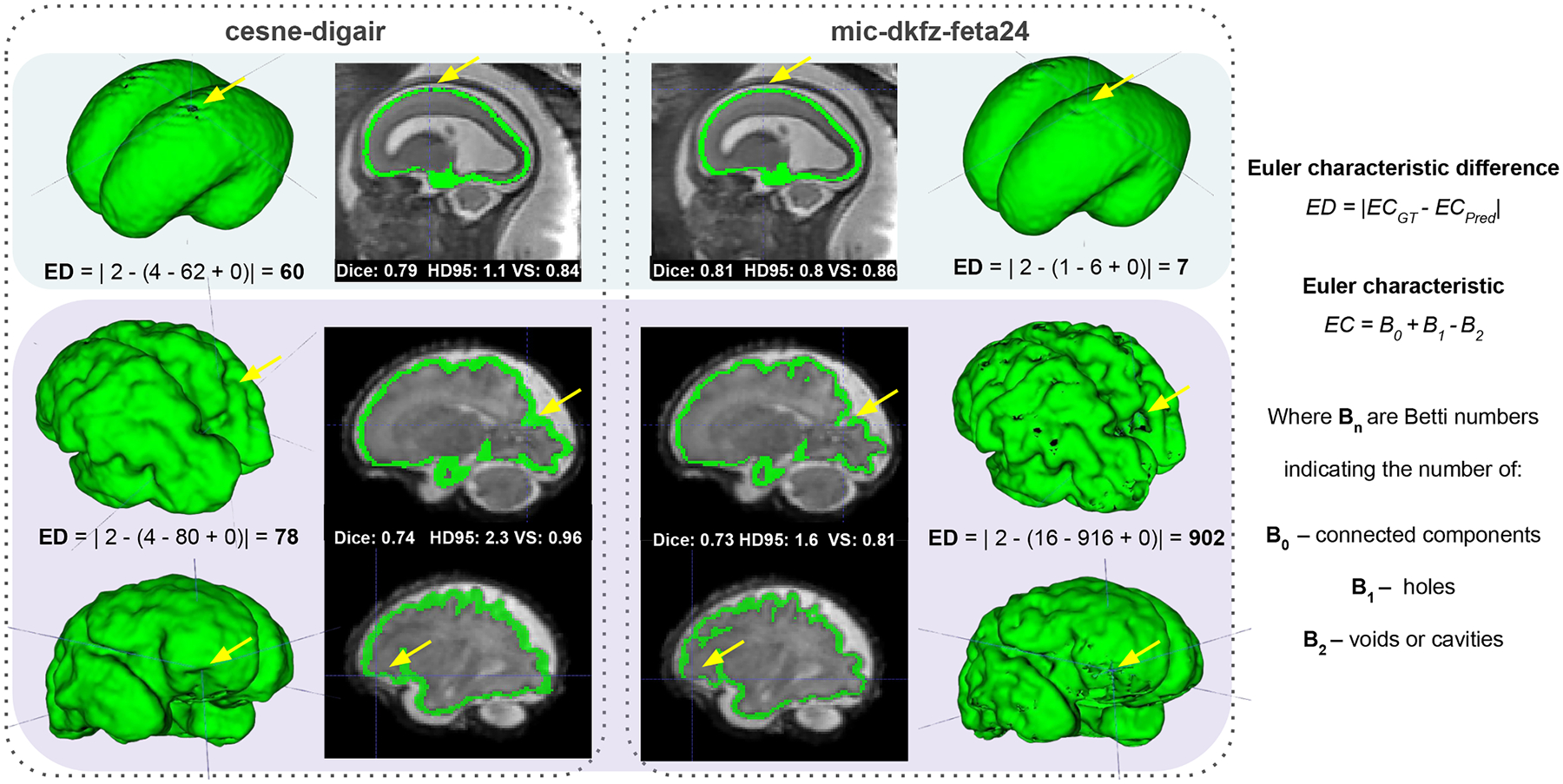
Segmentation results and reconstructed cortical GM surfaces for two representative fetal cases from cesne-digair and mic-dkfz-feta24, visualized using ITK-SNAP (Yushkevich et al., 2006). The top row (fetus at 22 weeks GA) illustrates that higher Dice scores sometimes correspond to smaller topological errors. However, the bottom two rows (fetus at 30 weeks GA) demonstrate significant topological issues (e.g., holes, fragmentation) in the mic-dkfz-feta24 surface, despite comparable Dice and HD95 values. Yellow arrows point to the same region on the image and the GM surface where strong topological errors can be seen. This underscores the need for additional topological and structural metrics, such as ED, to comprehensively evaluate segmentation quality, as metrics like Dice or HD95 alone are insufficient to capture topological accuracy.

Fig. 6 further illustrates the added value of topological metrics. In a comparative example, mic-dkfz-feta24 achieves similar Dice and lower HD95 scores but poorer ED and VS, suggesting that voxel-level agreement alone may not suffice for tasks requiring topologically accurate surfaces, such as morphological analysis.

##### Per-tissue and condition analysis.

Extended performance results split by site, tissue label, and pathology status are available in supplementary materials (sections A3, A4, and A5, respectively).

#### 3.3.2. Biometry ranking

##### Performance across sites and measurement.

**Fig. 7.**
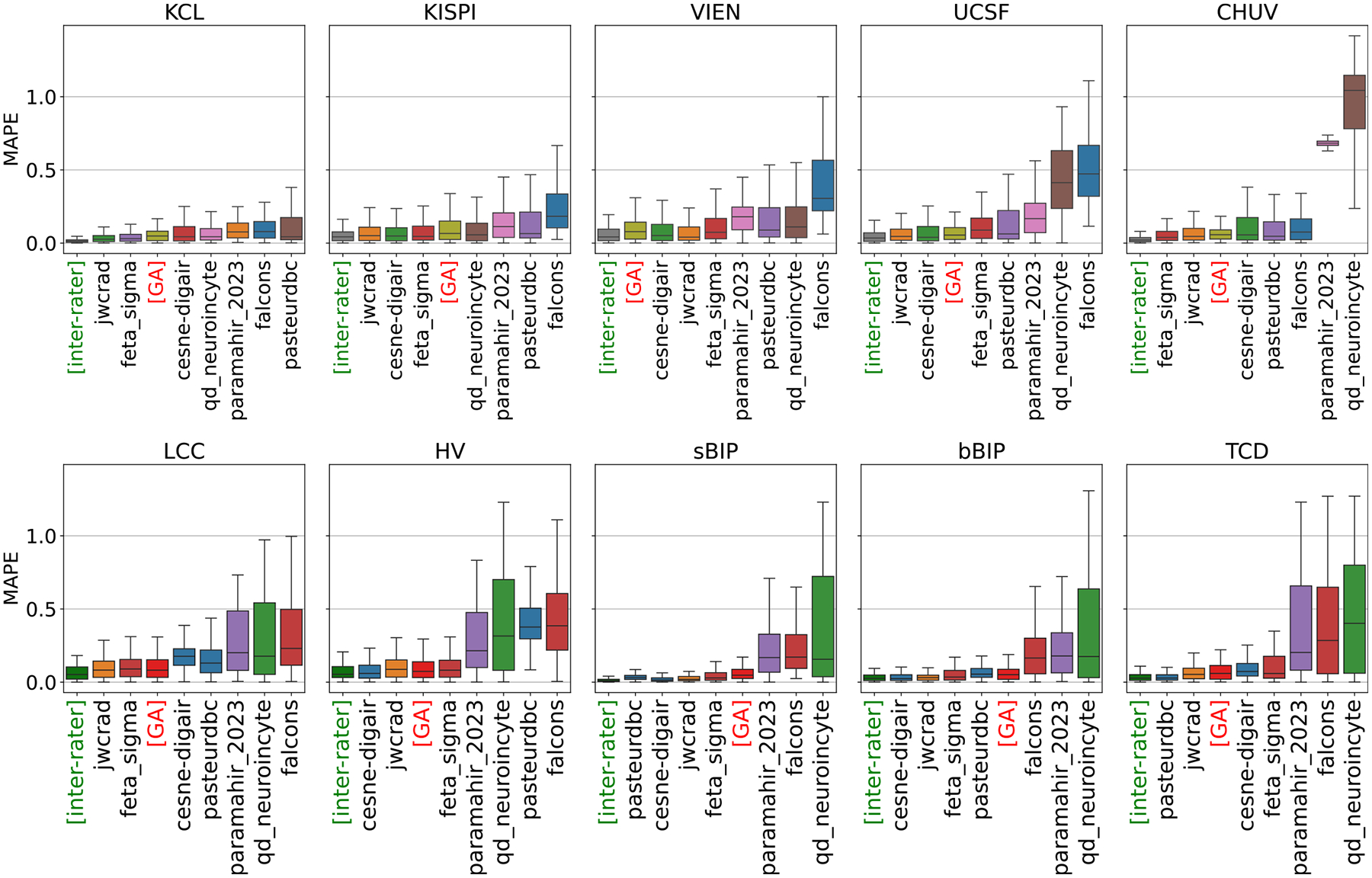
Biometry results per site (top) and label (bottom) for all teams participating in the Task 2 together with the GA baseline model ([GA]) and the inter-rater variability [inter-rater]. Teams are sorted in ascending order for each subplot independently, based on their mean MAPE for a given site or label.

Fig. 7 summarizes model performance per site and biometric measurement, with detailed values available in the supplementary materials section A6.

VIEN was the most challenging site, where no method outperformed the **[GA]** baseline (MAPE: 0.106±0.112), including the best-performing teams cesne-digair and jwcrad, which reached similar error levels. In contrast, KISPI emerged as the least challenging, with all three top teams exceeding the baseline. Across KCL, UCSF, and CHUV, only two teams per site (out of the top 3: jwcrad, feta_sigma, cesne_digair) achieved better-than-baseline performance. Measurement-wise, LCC, HV, and TCD were consistently more difficult, with HV and LCC showing the highest MAPE across all teams and raters. In contrast, sBIP and bBIP were among the best estimated. Notably, only jwcrad surpassed the baseline across all measurements, while a few others, including feta_sigma and pasteurdbc, did so on selected metrics.

Although multiple teams performed comparably on individual metrics, the clear winner in the ranking (see Table 5) was jwcrad, demonstrating consistent superiority across both site and measurement variations.

##### Robustness in pathological vs. neurotypical condition.

**Fig. 8.**
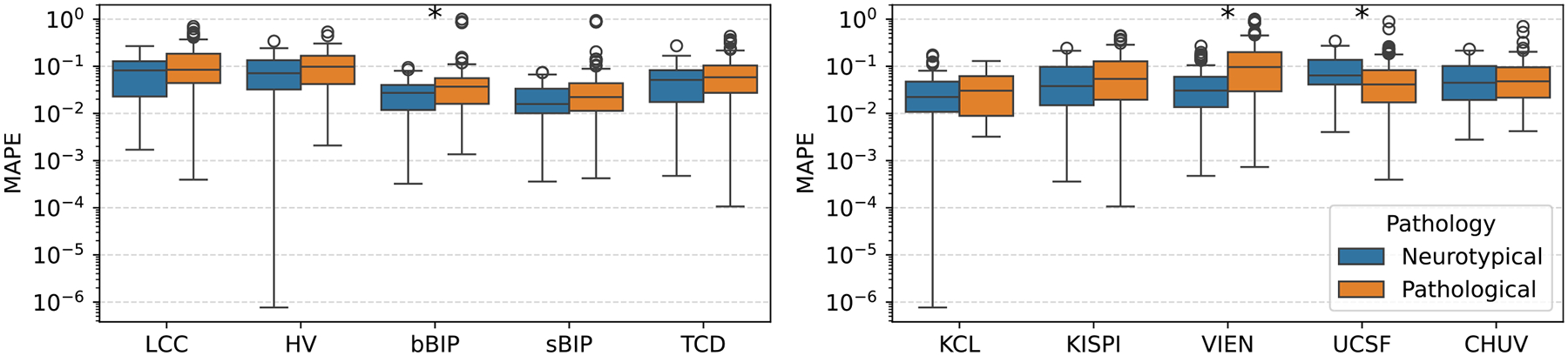
Biometry results for healthy and pathological subjects across labels and sites for the winning team jwcrad. Asterisks above the boxplot indicate statistically significant differences between the two groups (p <0.05, Mann-Whitney test).

To assess model generalizability, we compared biometry performance between neurotypical and pathological brains (see Fig. 8). While most measurements did not reveal statistically significant differences between groups, bBIP showed better accuracy in the healthy cohort, particularly at VIEN. Conversely,

UCSF results suggested slightly better performance for pathological subjects. Additional results in Appendix A6 further confirm the trend observed in Fig. 8 across teams, and highlight generally consistent changes with respect to the [GA] baseline across sites, anatomical structures and conditions.

In summary, the best-performing method, jwcrad, came within 9% of expert agreement for some measurements (e.g., TCD). However, for others like bBIP, its results differed from the expert range by as much as 60%. This reveals important gaps where automated biometry methods still fall short, especially in pathological cases.

### 3.4. Segmentation performance across challenge editions (2021, 2022 and 2024)

**Fig. 9.**
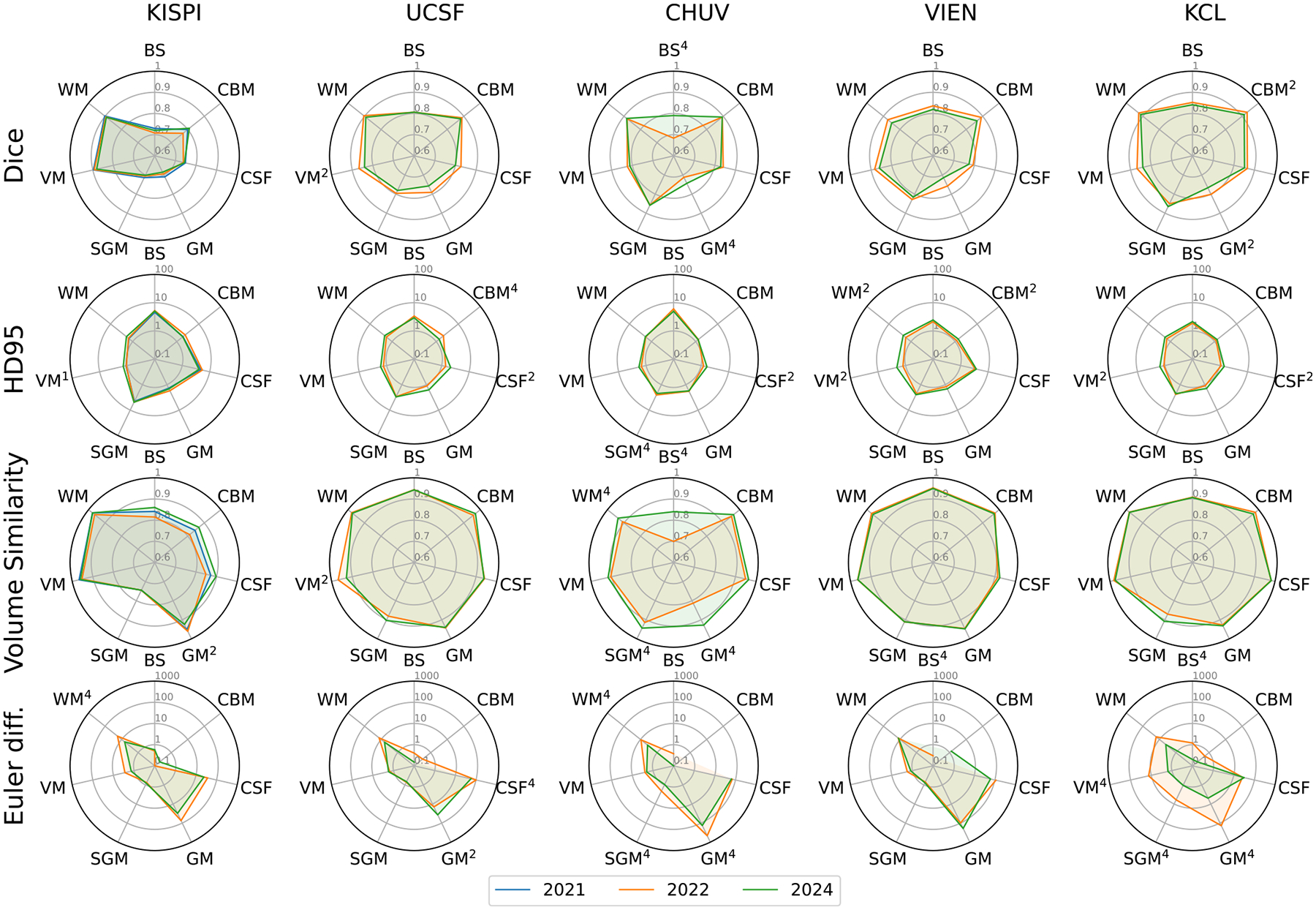
Segmentation performance improvement over the three editions of the FeTA Challenge. The superscript number above each label indicates whether the performance for a particular year and site-label-metric combination was statistically significantly better compared to all other available years. A superscript “2” indicates that the results for 2022 were the best, while a superscript “4” indicates that the results for 2024 were the best.

Over the three editions of the FeTA challenge, the segmentation task has expanded both in terms of dataset size (from 40 to 180 test cases) and site diversity (from 1 to 5 imaging centers). To evaluate progress over time, we compared segmentation performance across the years 2021, 2022, and 2024, focusing on common testing sites. Table 6 summarizes aggregated metrics for the top-performing teams each year (cesne-digair for 2024, FIT_1 for 2022 and NVAUTO for 2021), and Fig. 9 provides a visual overview of mean scores per label and site across years, with markers for statistically significant differences.

#### KISPI split (2021–2024).

This is the only site included in all three editions. No statistically significant improvement over the years was observed across the tracked metrics in the 2021 edition: Dice (0.79±0.16 → 0.77±0.18 → 0.78±0.15), HD95 (2.81±3.43 → 3.17±4.16 → 2.95±2.86), and VS (0.89±0.16 → 0.87±0.18 → 0.89±0.14). The only statistically significant change occurred in the VS metric for the GM label between 2021 and 2022 (0.96 → 0.94) and for the mean ED between 2022 and 2024 editions (18.98±54.56 → 9.21±17.84).

#### Other sites (2022–2024).

For sites such as CHUV, KCL, UCSF, and VIEN, which were included in both 2022 and 2024, only CHUV showed significant improvements for Dice and VS, while performance for other sites remained stable or decreased. Notably, ED improved substantially for CHUV, KCL, and KISPI. However, this improvement is influenced by the inclusion of ED in the 2024 ranking, which favored algorithms with better topological performance.

Overall, although methods have become more sophisticated and the data more diverse, performance has not consistently improved across editions.

### 3.5. Domain shifts evaluation

#### 3.5.1. Impact of image quality on performance

**Fig. 10.**
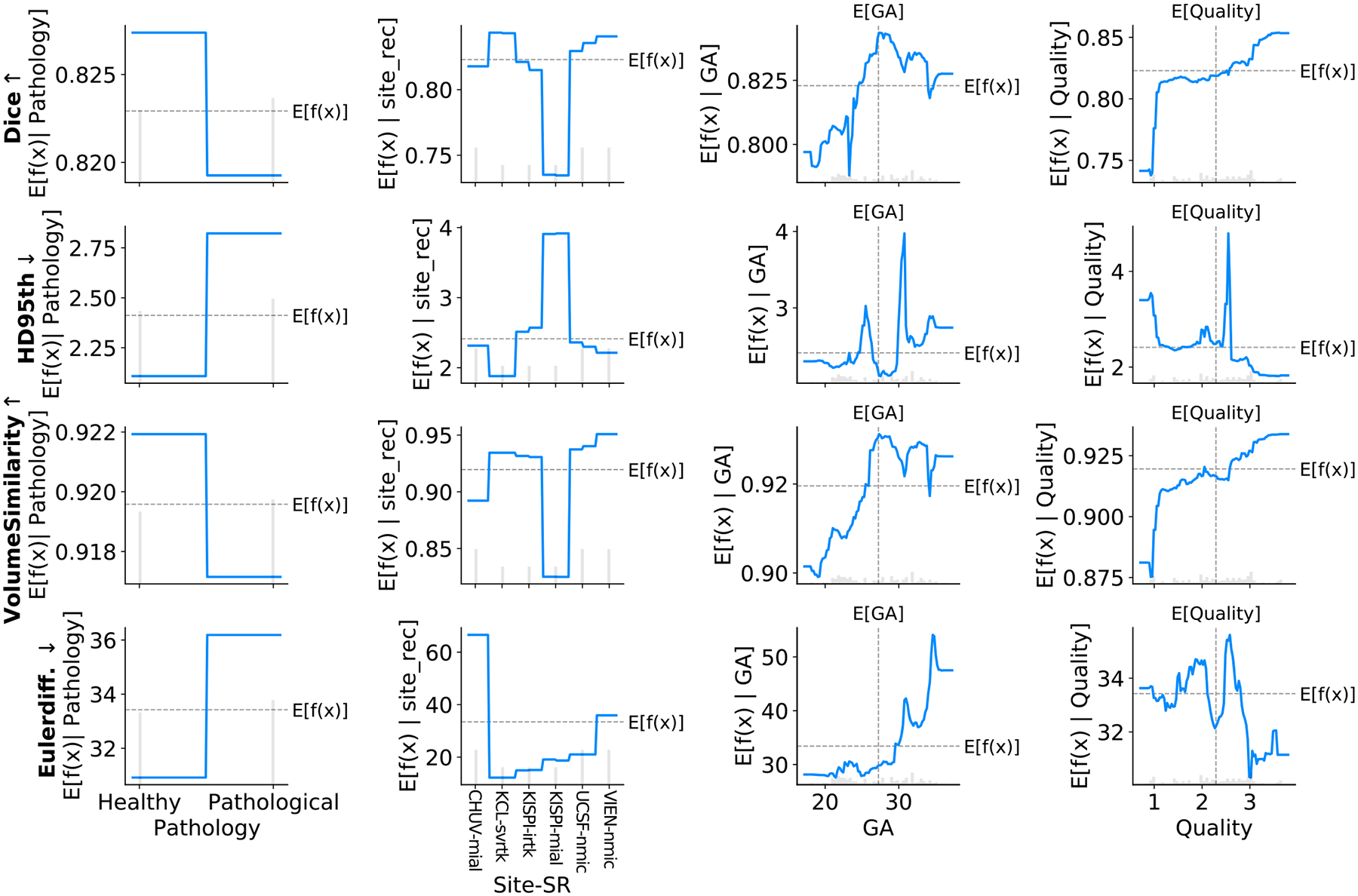
**Conditional mean** plots for Dice, HD95, VS and ED, for different domain shift factors: pathology, site and SRR, GA and image quality. The conditional mean shows how a given metric deviates from the global expected performance (E[f(x)∣) when a specific variable is used for conditioning.

The impact of image quality on model performance as determined by computing the conditional mean across quality ratings (E[f(x)∣Quality]) is shown in the right-most column in Fig. 10. We see a clear effect of image quality on Dice, with a generally increasing Dice with the increasing image quality, amounting to a change from 0.75 Dice on average for the lowest quality data (with scores close to 1) and an average quality close to 0.85 for the highest quality data. Results using HD95 and VS generally align with the ones from Dice, except for GA and quality. The relationship is, however, not as clear for ED, although best quality images tend to yield the smallest ED.

A more detailed analysis of the correlation between quality and the difference scores in the supplementary materials A7 showed a generally high Pearson correlation between quality and Dice (*r* = 0.5–0.7) for all sites except KISPI-mial (*r*=0.4) and USCF-nmic (*r*=0.06 – no correlation). The same trends, although weaker, were observed for HD95 and VS, except CHUV-mial and HD95, which had virtually no correlation (*r*=0.05). Results for ED showed no clear pattern, and larger correlations (*r*=0.3–0.4) were not statistically significant.

#### 3.5.2. Relative contribution of domain-shift sources

Fig. 10 displays the conditional means across four key variables (data quality, GA, pathology and site-SRR). The analysis revealed a pronounced site-SRR effect: for example, the KISPI-mial site produced notably lower Dice scores, whereas the CHUV-mial site was associated with higher ED values. In addition, gestational age (GA) significantly affected both Dice and ED scores. Similar trends can be found in the supplementary materials (appendix A7), although HD95 scores appear to be less influenced by GA.

**Fig. 11.**
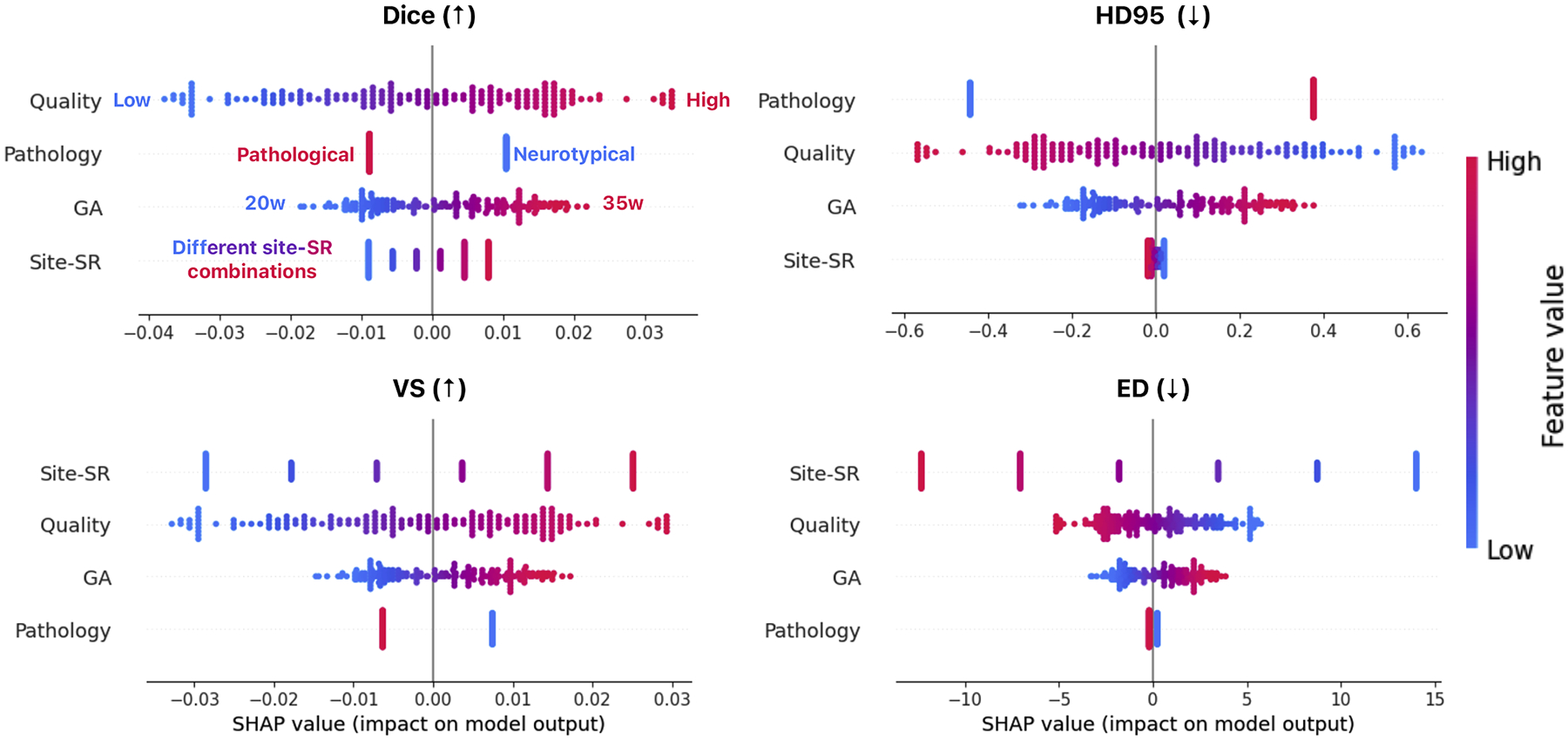
**SHAP value distributions** showing the average deviation from the unconditional expectation for the four segmentation metrics. They quantify how different factors contribute to each metric’s final score. Dot colors represent variable values: blue indicates lower values (e.g., low GA or quality), while red indicates higher values. For categorical variables (Site-SRR, pathology), colors have no specific meaning. Because SHAP values are additive, the expected metric for a subject can be obtained by summing the SHAP values of their site, GA, quality, and other conditions: for VS, a subject from VIEN-nmic (dark red), of high-quality, 35w GA and neurotypical would have in *average* a VS of 0.085 = 0.025 (site-SRR) + 0.03 (quality) + 0.02 (GA) + 0.01 (pathology) above the unconditional expected VS.

Fig. 11 presents a SHAP analysis for all metrics across the variables mentioned above. Note that this SHAP values here only explain part of the variance observed. As SHAP values are obtained from a random forest predicting the target metric from the factors, they are limited by how well the models capture the data. A five fold cross-validation showed that the fitted random forests explained the variance of 63 ± 11% of Dice, 38 ± 18% of HD95, 71 ± 12% of VS and 66 ± 8% of ED. Results of HD95 should then be interpreted with caution as the explained variance is only around 38%. Overall, the SHAP analysis summarizes how these factors influence the Dice and ED scores: image quality generally has the largest impact, followed by site-SRR. Although the pathological status of a subject generally has a small effect, we observed that severely pathological cases often have lower GA, which might introduce confounding. The plot’s color coding further confirms that higher image quality and GA are associated with increased Dice scores—for example, poor quality data may result in about −0.05 Dice, compared to an average of +0.03 Dice for good quality data.

## 4. Discussion

### 4.1. FeTA 2024 results and ranking

The multi-site, multi-task design of this challenge offered a unique opportunity to evaluate the progress and robustness of fetal brain image analysis algorithms. We summarize the main observations below.

#### Segmentation task.

As in FeTA 2022, the top three teams all used 3D U-Net or nnU-Net architectures, leveraging the strong inductive biases of CNNs for volumetric data while Transformer-based designs (e.g., Swin Transformer used by cemrg and qd_neuroincyte) underperformed, highlighting the particular **benefits of CNN based approaches in a low-data regime like the FeTA challenge**. All 16 teams employed **data augmentation**. Standard spatial and intensity augmentations (e.g., gamma transformation, noise, blur, affine transformation) were almost universal, while several teams applied MRI-specific augmentations to simulate fetal MRI artifacts such as k-space ghosting, spikes, and motion. Some went further, with notable gains in ranking. For example, vicorob (3rd place) combined motion and bias-field simulation with SynthSeg-inspired image synthesis, whereas the winning team, cesne-digair, generated anatomically plausible pathological cases via deformable registration between healthy and pathological brains. **Augmenting both image appearance and anatomy was crucial for strong performance** in our multi-center setting, given the limited data from some acquisition sites and the diverse, numerous pathological cases in the testing dataset. External data, or **models trained on external datasets, did not substantially improve performance**, as two of the top three methods, including the winner, relied solely on the challenge images and segmentations.

**Ensembling was common among high performers**, combining cross-validation models as well as models with different architectures or trained on different datasets and augmentation schemes. Notably, cesne-digair applied a denoising autoencoder as a post-processing step in their ensembling scheme, achieving a 50% improvement in Euler Difference over the second-best team. This highlights that **topological correctness in segmentation remains underexplored**, with many models not explicitly prioritizing it, and that even a simple approach like a DAE can yield substantial gains.

#### Topology metric is a valuable add-on.

In the brain tissue segmentation task, the introduction of the topology-aware metric provided meaningful complementary insights beyond traditional overlap-based measures. Despite the architectural diversity and growing methodological complexity of the submitted approaches, the performance differences among the top teams were minimal, with Dice scores showing tight clustering, suggesting that gains in segmentation accuracy may be reaching a plateau. Differences in team rankings across evaluation metrics (Dice, HD95, VS, ED) highlight the need to consider complementary metrics beyond voxel-wise overlap. Introducing ED as a ranking metric provided a more nuanced assessment of the segmentation quality. This is reflected in Table 6, where we see a marked improvement in ED. While teams did not specifically optimize their models for topological consistency, the new ranking scheme allowed us to discriminate between methods that otherwise had very similar performances (Table 4). Evaluation noise, where performance variations across testing sets is larger than the difference across top-performing methods, is a well-known problem in medical imaging challenges (Varoquaux and Cheplygina, 2022), and the introduction of an additional ranking metric allowed for selection methods with desirable properties. Further validation on clinical tasks leveraging surface extraction (Clouchoux et al., 2011; Yehuda et al., 2023) would be needed to truly see the potential of encouraging topological consistency in the FeTA challenge.

#### Low-field MRI tissue segmentation quality is encouraging.

The newly introduced 0.55T data from KCL provided an unexpected insight: it consistently achieved the highest segmentation accuracy across all sites. However, it is important to note that, to reduce domain shifts, we retrospectively selected high-quality reconstructions using a version of SVRTK specifically tailored for low-field MRI data (Uus et al., 2020, 2024). This careful case selection, paired with a very recent SRR pipeline, might have positively biased the performance for this cohort. As such, performance on more challenging low-field cases remains to be fully assessed. Nevertheless, these results are encouraging for two reasons: progress in SRR pipelines (Uus et al., 2024; Xu et al., 2023) means that more challenging cases will be successfully reconstructed, and that image quality will generally increase (Sanchez et al., 2024a; Uus et al., 2023b). Low-field MRI systems hold significant promise for expanding access to prenatal imaging, particularly in low- and middle-income countries. When combined with good image quality and advances in automatic fetal exam planning (Neves Silva et al., 2024), they could meaningfully enhance prenatal care in resource-limited settings.

#### Biometry task.

Seven teams participated in this task, **all leveraging segmentation outputs** but employing diverse estimation strategies, direct biometry regression, 3D landmark coordinate prediction, or landmark heatmap segmentation, reflecting the absence of a standard approach. The top three teams used different architectures (CNN, U-Net, Transformer), yet **all relied on 3D models**.

The winning pipeline, jwcrad, **cropped and masked images using segmentation outputs** before predicting landmark heatmaps, allowing the biometry model to focus solely on the relevant region of interest. They were the only team to outperform the gestational-age baseline, which many others failed to surpass. Across methods, bBIP and sBIP were estimated most accurately, whereas HV and LCC remained challenging. **External data had limited impact in this task too**, with two of the top three teams relying exclusively on the challenge dataset.

#### Automated biometry needs strong baselines to ensure meaningful progress.

To reflect current clinical practice and bridge the gap between routine 2D fetal brain assessments and emerging 3D imaging techniques, we introduced a new biometry task focused on 2D brain measurements, key clinical indicators traditionally used to assess fetal neurodevelopmental status (Tilea et al., 2009; Lamon et al., 2024). One of the striking results of this first edition is that most submissions did not manage to outperform a simple model, that predicts biometry solely based on gestational age, completely ignoring image information.

This can be partly attributed to the strong correlation between gestational age and brain growth for the selected measurements (Tilea et al., 2009), the relatively small and partially missing training set compared to the model complexity, and the fact that all submitted approaches relied on full 3D segmentations for biometry estimation, introducing potential error propagation, especially for small or anatomically complex structures such as the corpus callosum or vermis. In contrast, clinicians typically assess these metrics using carefully selected 2D slices aligned with standard anatomical planes. Although we provided participants with both the transformation matrices used to reorient images into the radiological annotation space and the 3D landmark coordinates used to compute biometry, no team fully exploited both of these resources during training. This disconnect may have hindered model performance, as these elements directly reflect how the annotations were originally derived. Models may have performed better by mimicking clinical workflows: detecting relevant anatomical planes (mid-sagittal for LCC and vermis height; transventricular/trans-thalamic for BPD; transcerebellar for TCD-using 3D pose/plane proposal networks or atlas-guided reformatting) and applying lightweight 2D estimators or landmark heatmap predictors, rather than relying on indirect 3D volume processing requiring larger, less targeted models. In ultrasound, plane estimation is a commonly used pre-processing step for automated biometry (Avisdris et al., 2022; Bano et al., 2021), and more recently, similar strategies in MRI have shown promising results as a foundation for accurate measurement (Luis et al., 2025).

Moreover, evaluating 2D-derived metrics from 3D segmentations may not fully leverage the strengths of super-resolution reconstruction. Introducing volumetric or surface-based biomarkers-such as cortical surface area or gyrification indices-could better capture the benefits of 3D data and topology-aware segmentation. However, clinical practice still relies on 2D biomarkers measured on 2D slices from HASTE acquisition. The development of more specific volumetric biomarkers is still in its infancy and will require further work for the validation and integration of SRR and automatic segmentation tools (Khawam et al., 2021; Sanchez et al., 2025a).

Fetal brain biometric measurements used in this study are known to scale tightly and approximately monotonically with GA, which explains why a simple univariate GA regressor was able to capture a large portion of the variance. However, despite the strong performance of this baseline, it remains fundamentally limited by its inability to account for individual anatomical variation, atypical development, or pathological deviations. To overcome these limitations, future biometry models could incorporate gestational age as a prior and learn image-driven residuals, allowing them to retain the robustness of GA-based predictions while adapting to subject-specific features. In addition, incorporating uncertainty estimation to trigger human review or to fall back to the GA baseline when confidence is low could help identify unreliable predictions and guide clinical users toward more trustworthy outputs (Fidon et al., 2024). Such models would benefit from focusing on clinically relevant strategies-such as anatomically informed plane selection or structure-aware localization-that better reflect radiological practice.

### 4.2. FeTA challenge in perspective

#### FeTA across the years.

A retrospective analysis of FeTA challenge results over the years revealed no statistically significant improvements in performance metrics, with the exception of ED at two out of five sites. Similarly, no notable improvement was seen at the label level, with GM, SGM, and BS consistently remaining the most challenging structures to segment. GM is particularly difficult due to its very thin appearance in fetal brains, where partial volume effects and complex surface morphology make it especially prone to topological segmentation errors, leading to significantly higher ED values compared to other labels. Moreover, both GM and SGM have inherently low tissue contrast in MRI, making them harder to distinguish accurately (Prayer et al., 2006). These challenges are further illustrated in Supplementary Materials A9.1, which provide qualitative examples showing that most segmentation errors occur in regions corresponding to GM, SGM, and BS. This outcome is not entirely unexpected, as most top-performing teams relied on similar 3D architectures-primarily 3D U-Net (Çiçek et al., 2016) and nnU-Net (Isensee et al., 2018)-enhanced with extensive data augmentation and model ensembling. These findings suggest that incremental architectural modifications or model engineering alone are unlikely to yield substantial gains, aligning with trends observed in other challenges where U-Net-based approaches often outperform more complex alternatives (Eisenmann et al., 2023). While these techniques help mitigate certain domain shifts related to scanner differences or pathological variations, some cases remain persistently difficult across all methods. Addressing these harder cases may require deeper domain expertise and a shift toward a more data-centric approach, prioritizing data quality, annotation consistency, and dataset diversity as core components of model development (Sambasivan et al., 2021; Zha et al., 2023).

#### Sources of domain shifts.

Domain shifts are widely recognized as a key challenge for deep learning methods in medical imaging (Dockès et al., 2021; Wiles et al., 2021; Richiardi et al., 2025), yet the specific sources of these shifts are rarely disentangled. In our analysis, though not causal, we observed that image quality had the strongest impact on generalization performance: moving from the lowest to the highest quality levels resulted in an average Dice score difference of approximately 0.10. In contrast, gestational age had a more modest effect, influencing Dice scores by about 0.05, while the scanning site contributed a difference of around 0.075 between the best- and worst-performing centers. Interestingly, pathology was the least influential factor, accounting for only about 0.008 in Dice variation. Additionally, because Dice is known to be biased toward larger structures (Maier-Hein et al., 2024), we also evaluated performance using a normalized Dice metric that accounts for label volume (Raina et al., 2023). As detailed in the supplementary materials section A8, the normalized Dice scores yielded rankings nearly identical to those based on standard Dice, indicating that structure size did not significantly distort the comparative performance of the reviewed algorithms, suggesting that while the size bias exists, its effect was uniform across methods.

Our results show that, despite pathological cases making up only about one-third of the training data, models were still able to generalize to pathological examples in the test set. While performance was slightly lower for pathological subjects in some datasets compared to healthy subjects, submitted models demonstrated the ability to correctly handle both healthy and pathological data. Given the rarity and wide variability of fetal pathologies (Attallah et al., 2019), expanding pathological datasets-whether through additional real cases or synthetic data (Dannecker et al., 2024; Kaandorp et al., 2025b)-will be crucial to narrowing this performance gap and improving overall model robustness, which is an important step toward real-world clinical deployment.

Overall, our findings suggest that technical and acquisition-related factors may play a more significant role in out-of-domain generalization than subject-level clinical variables. Still, further causal investigations (Castro et al., 2020) are needed to confirm these patterns and to avoid misinterpretation due to confounding factors.

### 4.3. Roadmap for future advancements in fetal brain MRI analysis

While many proposed solutions appear to be reaching a performance plateau, model-centric innovations still play an important role. That said, incorporating domain-specific augmentations and auxiliary learning objectives may lead to more impactful improvements than simply refining model architectures. For example, enforcing *topological consistency* within the loss function, as demonstrated by de Dumast et al. (2022), Li et al. (2023), Lux et al. (2024)-can help maintain anatomical plausibility in the predictions. An additional priority for future editions of the challenge is the systematic integration of uncertainty quantification (Maier-Hein et al., 2024), such as predictive intervals, confidence maps, and calibration measures, to better capture model reliability and support safer clinical translation. Incorporating these outputs, whether as optional or required components, would enable participants to report not only target values but also the associated confidence, which is essential in settings with limited-quality SRR data. Several studies (Zenk et al., 2025; Molchanova et al., 2025) have demonstrated the utility of uncertainty-aware models for quality control in medical image segmentation.

Beyond model architecture, data-driven strategies hold substantial potential for improvement. A notable limitation of current solutions is the relatively modest use of *external data*, which has been largely limited to *healthy* subjects from datasets like dHCP or fetal brain atlases (Gholipour et al., 2017; Uus et al., 2023a; Price et al., 2019). Leveraging broader, more diverse datasets, especially those capturing rare or pathological conditions, could support more robust and clinically useful models, though curating and annotating pathological datasets is a huge endeavor. Another important future direction is to provide participants access to the raw, pre-SRR stacks. This would allow a systematic assessment of how SRR choices affect downstream segmentation quality and would enable teams to integrate reconstruction steps directly into their proposed solutions. The broader scientific question of how different SRR strategies, artifact profiles, and reconstruction parameters shape segmentation robustness remains open, and understanding these interactions will be essential for advancing both methodological development and clinical applicability.

Manually segmenting the fetal brain is a time-consuming and tedious task, susceptible to inter-rater variability (Payette et al., 2021), and the FeTA challenge data are not exempt from this issue. When comparing model performance to inter-rater variability, we observe that top-performing teams-achieving Dice scores around 0.82, HD95 around 2.2, and VS around 0.92-are approaching the best observed human agreement levels, previously estimated on a subset of data as 0.73 ± 0.15 for Dice, 3.45 ± 2.34 for HD95, and 0.86 ± 0.10 for VS (Payette et al., 2023). This raises the intriguing possibility that some predictions may be more faithful to the underlying anatomy than the ground truth annotations, potentially leading to penalization of high-performing models (Valabregue et al., 2024a,b).

A promising direction to address the limitations of data diversity and annotation availability is data synthesis (Zalevskyi et al., 2024), particularly for generating rare or pathological fetal brain appearances. Recent work (Dannecker et al., 2024; Liu et al., 2024; Kaandorp et al., 2025a; Plana et al., 2025) has highlighted the potential of synthetic data to augment training and improve sensitivity to abnormal anatomy. Moreover, the strong influence of image quality on generalization performance underscores the need for better modeling of artifacts specific to fetal brain SRR pipelines (Sanchez et al., 2024a).

The ultimate goal of automated segmentation and biometry algorithms is their integration into both research and clinical practice. However, real-world deployment must also consider regulatory and ethical challenges. While frameworks like the EU AI Act (EU., 2024) promote trustworthy AI, they often lag behind rapid technical progress. Bridging this gap requires aligning methodological advances with evolving legal and clinical standards, while also addressing fairness by evaluating model performance across variables such as sex, race, and across different underrepresented populations (Lekadir et al., 2025).

## 5. Conclusion

The FeTA 2024 challenge provided a valuable opportunity to evaluate the progress made in fetal brain segmentation since previous editions and to expand the scope toward new, clinically relevant tasks such as biometry. Our additional validation using the Euler difference metric showed that some existing methods can already produce topologically consistent segmentations. However, achieving this consistency more reliably, particularly through improved segmentation losses, remains an open area for further development. Likewise, the successful application of models to low-field data, with surprisingly strong performance, highlights both the advancements in recent super-resolution methods and the models’ capacity to generalize across diverse imaging settings.

In the biometry task, this first edition offered key insights, particularly on the importance of providing simple baseline models to guide participants. It also led to the emergence of a promising approach for automated biometry prediction.

As the field of fetal brain MRI analysis continues to evolve, FeTA 2024 emphasizes the need not only for more powerful and innovative models but also for building reliable and generalizable tools that can support real-world clinical adoption.

## Supplementary Material

1

Supplementary material associated with this article can be found, in the online version, at 10.1016/j.media.2026.103941

## Figures and Tables

**Table 1:** FeTA 2024 datasets properties. *Nn* - number of neurotypical subjects, *Np* - number of pathological subjects.

Used for	Testing domain	Institution	Scanner	N	SRR method	SRR res. (mm^3^)	TR/TE (ms)	GA (weeks)	N_n_/N_p_
**Training**	In domain	KISPI	GE Signa Discovery MR450/MR750 (1.5T/3T respectively)*^a^	80	MIALSRTK (40) IRTK-simple (40)	(0.5)^3^	2000–3500/ 120 +	20–34.4	31/49
		Vienna	Philips Ingenia/Intera (1.5T) Philips Achieva (3T)*	40	NiftyMIC	(1.0)^3^	6000–22000/80–140	19.3–34.4	33/7
**Testing**	In domain	KISPI	GE Signa Discovery MR450/MR750 (1.5T/3T respectively)*	40	MIALSRTK (20) IRTK-simple (20)	(0.5)^3^	2000–3500/ 120 +	21.3–34.6	15/25
		Vienna	Philips Ingenia/Intera (1.5T) Philips Achieva (3T)*	40	NiftyMIC	(1.0)^3^	6000–22000/80–140	18.1–35.5	20/20
	Out of domain	CHUV	Siemens MAGNETOM Aera (1.5T)	40	MIALSRTK	(1.125)^3^	1200/90	21.0–35.0	25/15
		UCSF	GE Signa Discovery MR750/MR750W (3T)	40	NiftyMIC	(0.8)^3^	200–3500/100+	20.0–35.1	8/32
		KCL	Siemens MAGNETOM Free.Max (0.55T)	20	SVRTK	(0.8)^3^	2500/106	21.0–35.0	15/5

“+” indicates the minimum TE value.

a * The training dataset contained data from both 1.5T and 3T scanners. However, which cases belonged to which scanner were not provided to the participants as it was part of the data anonymization process. Therefore, the breakdown of number of cases per scanner is not provided here.

**Table 2:** Summary of the algorithms submitted for the fetal brain tissue segmentation task.

Team name	Model Architecture	DL Framework	Dim	Data Augmentation	Cross-Validation	External Data	Ensembling	Original Aspects
cemrg	Hybrid Cross Attention Swin Transformer and CNN	PyTorch, nnUNet	3D	Horizontal Flipping, Vertical flipping, scaling, normalization	5-fold	No	No	The Cross Attention Transformer (CAT) block design.
cesne-digair	3D UNet	MONAI	3D	Deformable (SyN) registrations between couples of neurotypicall and pathological scans from the preprocessed training dataset. Skull-stripping with BOUNTI	Not specified	No	Use of models for post-processing	Denoising autoencoder for segmentation accuracy enhancement.
falcons	2D Attention Gated U-Net	TensorFlow	2D	Rotation, width/height shift, vertical/horizontal flip, zooming, brightness, gaussian noise, gaussian blurring	Not specified	70 images from dHCP)	Models with different architecture and (or) training data	Series of preprocessing steps including brain extraction, alignment, and non-uniform intensity correction. Ensembling of models trained on different orientations (axial, sagital, coronal)
feta_sigma	UxLSTMEnc, UNet	nnUNet	3D	Rotation, Scaling, Translation, Gaussian Noise, Mirror Transform.	5-fold	No	Models with different architecture and (or) training data	Use of UxLSTM and ensembling with nnUnet, Background masking.
hilab	nnU-Net	PyTorch, nnUNet	3D	Default nnU-Net augmentations, histogram equalization, differentiated probabilities for sample selection in random copy-paste augmentations, replication of challenging cases.	5-fold	No	Models with different architecture and (or) training data	Applying histogram equalisation to 3D images, differentiated probabilities for sample selection in random copy-paste augmentations, strategically replicating challenging cases in the training data. Enesemble of 5 models with different hyperparameters and pre-processing settings.
jwcrad	Residual-USE-Net	PyTorch, MONAI	3D	Rotation, scaling, translation, intensity shift, low resolution simulation.	5-fold	No	Model trained on differnt CV splits	Custom auxiliary loss function based on transformation consistency.
LIT	Attention UNet, nnUNet ResidualEncoderUNet	PyTorch, nnUNet	3D	Rotation, Scaling, Gaussian Noise, Gaussian Blur, Brightness Alteration, Contrast Adjustment, Low Resolution Simulation, Gamma Adjustment, Mirroring	6-fold	No	Model trained on differnt CV splits	Custom brain mask detection with Attention Unet
lmrcmc	nnUNet, SegVol	nnUNet, MONAI	3D	nnUNet: default, SegVol: flip, ScaleIntensity, ShiftIntensity, GibbsNoise, BiasField, KSpaceSpikeNoise and Affine augmentation; with SLAug.	Not specified	No	Models with different architecture and (or) training data	Ensemble of U-Net and a foundation model, use of the SegVol model in fetal brain segmentation.
mic-dkfz	U-Net nnUNet), U-Net with Residual encoder	nnUNet	3D	randomized; blur, gaussian noise, spatial (rotation, scaling, flipping), brightness, contrast, low-resolution simulation, gamma, sharpening, blank rectangle	5-fold	Yes (pre-training in MultiTalent)	Models with different architecture and (or) training data	Pretraining with MultiTalent on a collection of publicly available datasets. Ensemble of 3 nn-Unet configurations with different data augmentations.
paramahir_2023	3D UNet (segmentation), custom UNet-based (biometry)	MONAI	3D	Random Flipping, Random Rotation, Random Intensity Shifts	Not specified	No	No	Combination of segmentation and biometry prediction in a unified pipeline.
pasteurdbc	MedNeXt_L and nnUNet	nnUNet	3D	RandomScaling, RandomRoatation, RandomAdjustContrast, RandFlip	5-fold	Multi-modal multi-organ medical image datasets used in the pre-trained MedNeXt_L foundational model	Models with different architecture and (or) training data	Used additional datasets with CT and brain MRI images for model pre-training
qd_neuroincyte	Swin UNETR	MONAI	3D	Random sliding window, flipping, 1% gaussian noise, rigid rotation of ±25° around all axes, random shifting ± 5mm along all axes.	Not specified	No	Use of models for post-processing	Brain masking for vienna
unipd-sumaug	2D Swin-UMamba	PyTorch, nnUNetv2, Monai	2D	TorchIO transforms and GIN techniques, pair-wise co-registration, affine and rigid transforms using the Advanced Nomalization Tools.	5-fold	Model pre-trained on ImageNet	Model trained on differnt CV splits	Pretrained on imageNet repository and using GIN.
upfetal24	nnU-Net ResEncL	nnUNetv2	3D	Default nnU-Net augmentations; differentiated by specific data augmentations for each of the three models.	5-fold for config	dHCP and fetal atlasses	Models with different architecture and (or) training data	Data augmentation strategies and ensembling of models
vicorob	nnUNet	nnUNet, PyTorch	3D	Random bias field, motion artifacts, low-resolution simulation, SynthSeg-inspired T2w image synthesizer	3-fold	No	Model trained on differnt CV splits	SynthSeg-inspired T2w image synthesizer, Sharpness-Aware Minimization (SAM) optimizer

**Table 3:** Summary of the algorithms submitted for the biometry estimation task.

Team name	Architecture	Dimensionality	Original Aspects	External datasets	Framework/languange
qd_neuroincyte	SwinUnetr	3D	Relies on segmentation. Predict landmark heat maps only using the segmentation maps and then calculate biometry.	No additional data was used	Pytorch 2.2.2
cesne-digair	CNN	3D	Relies on segmentation. Predict the keypoints given the segmentation.	No additional data was used	PyTorch Version 2.4.0
jwcrad	Residual-USE-Net	3D	Relies on segmentation. Uses the segmentation maps to localize and preprocess the input images by masking and cropping the original 3D image. Predict landmark heat maps using the preprocessed images and then calculate biometry.	No additional data was used	PyTorch 2.2.2
pasteurdbc	MedNeXt_L nnUNet	3D	Use of a pre-trained foundational model.	Yes (for the pre-trained MedNeXt_L foundational model, multi-modal multi-organ medical image datasets)	
falcons	Attention Gated U-Net	2D	Relies on segmentation. Predict the biometry values directly	Yes (+ 70 images from dHCP)	Tensorflow(2.10.0) FMRIB Software Library(FSL 6.0), CIVET(2.1.0), Advanced Normalization Tools(ANTs), Scikit-learn (1.5.1)
feta_sigma	nnUNet, UxLSTMEnc	3D	Ensemble network of nnUnet and UxLSTMEnc.	No additional data was used	PyTorch
paramahir_2023	UNet	3D	Relies on segmentation. Predict the biometry values by regressing the U-Net features.	No additional data was used	PyTorch 2.3 -

**Table 4:** Segmentation ranking and average metrics. Metric values are reported as the mean across all labels. Models are sorted by final rank. HD95 is reported in millimeters.

Team	Dice		HD95		VS		ED		Mean rank	Final rank
	Rank	Value	Rank	Value	Rank	Value	Rank	Value		
cesne-digair	8	0.816	3	2.317	1	0.929	1	20.921	3.25	**1**
mic-dkfz-feta24	1	0.828	2	2.224	3	0.918	8	37.206	3.50	**2**
vicorob	2	0.825	1	2.187	2	0.920	11	41.293	4.00	**3**
feta_sigma	3	0.822	7	2.430	5	0.914	4	31.710	4.75	**4**
cemrg_feta	4	0.822	10	2.836	4	0.916	7	34.382	6.25	**5**
upfetal24	5	0.820	6	2.412	6	0.913	9	39.967	6.50	**6**
hilab	7	0.816	8	2.434	9	0.911	3	30.123	6.75	**7**
lit	10	0.808	5	2.391	8	0.911	10	40.085	8.25	**8**
lmrcmc	11	0.805	11	3.179	7	0.913	5	32.751	8.50	**9**
unipd-sum-aug	9	0.811	4	2.332	10	0.909	13	46.668	9.00	**10**
pasteurdbc	6	0.817	9	2.474	11	0.909	12	41.521	9.50	**11**
jwcrad	12	0.769	12	3.569	12	0.886	2	29.744	9.50	**11**
qd_neuroincyte	13	0.681	13	10.441	13	0.827	6	34.295	11.25	**13**
falcons	14	0.628	14	11.040	14	0.765	14	100.729	14.00	**14**
paramahir_2023	15	0.040	15	80.757	15	0.337	15	1416.515	15.00	**15**

**Table 5:** Metrics and ranking for the biometry estimation task sorted by the final MAPE.

Team	LCC		HV		bBIP		fBIP		TCD		Final MAPE	Final rank
	MAPE	Rank	MAPE	Rank	MAPE	Rank	MAPE	Rank	MAPE	Rank		
*[inter-rater]*	*9.59*	*	*8.04*	*	*3.28*	*	*1.49*	*	*4.89*	*	*5.38*	*
jwcrad	**11.15**	**1**	10.32	2	5.43	2	4.78	3	7.21	2	**7.72**	**1**
*[GA]*	*12.75*	*3*	*11.26*	*3*	*6.82*	*5*	*6.47*	*5*	*10.80*	*3*	*9.56*	*
cesne-digair	17.72	4	**9.82**	**1**	**4.02**	**1**	4.74	2	12.34	4	9.59	2
feta_sigma	12.59	2	11.55	4	5.74	3	5.54	4	13.66	5	9.76	3
pasteurdbc	20.47	5	43.48	7	6.51	4	**3.74**	**1**	**5.43**	**1**	15.83	4
paramahir_2023	28.48	6	29.35	5	26.13	7	25.46	6	30.78	6	28.03	5
falcons	34.88	8	46.25	8	24.62	6	28.13	7	36.72	7	34.09	6
qd_neuroincyte	32.78	7	42.84	6	38.41	8	37.83	8	47.92	8	40.07	7

[GA] and [inter-rater] entries do not represent participating models, thus their rank is marked as *.

**Table 6:** Mean and standard deviation (mean±std) for different metrics across years and splits over all labels.

Year	Site	Dice	HD95	VS	ED
2021	KISPI	0.79±0.16	2.81±3.43	0.89±0.16	not estimated
2022	CHUV	0.81±0.09	2.33±1.68	0.88±0.10	77.43±168.91
	KCL	0.87±0.05	1.46±0.57	0.95±0.05	28.61±53.89
	KISPI	0.77±0.18	3.17±4.16	0.87±0.18	18.98±54.56
	UCSF	0.84±0.06	2.02±1.44	0.95±0.05	18.73±44.52
	VIEN	0.84±0.08	1.87±1.46	0.95±0.06	32.20±67.05
2024	CHUV	**0.83±0.06** *	2.23±1.40	**0.93±0.06** *	**29.10±51.56** *
	KCL	0.86±0.05	1.69±0.52	0.95±0.04	**6.26±13.21** *
	KISPI	0.78±0.15	2.95±2.86	0.89±0.14	**9.21±17.84** *
	UCSF	0.82±0.07	2.13±1.31	0.94±0.05	14.57±25.90
	VIEN	0.81±0.09	2.27±1.69	0.95±0.05	38.13±93.48

Bold and * highlight the years that have statistically significant improvement in the values compared to the previous year. ED was not estimated in FeTA 2021.

## Data Availability

A subset of the challenge dataset used for benchmarking is publicly available on Synapse (Payette and Jakab, 2021), as described in the main manuscript. All submitted algorithm implementations are provided as Docker images and can be accessed on our DockerHub page at https://hub.docker.com/u/fetachallenge2024. The code used to run inference for all submitted Docker containers is available in the challenge’s GitHub repository at https://github.com/fetachallenge/fetachallengesubmission. The evaluation code used to compute all reported metrics is publicly accessible at https://fetachallenge.github.io/pages/Evaluation.

## Footnotes

1 Within the training data of FeTA, only the dataset from the University Children’s Hospital Zurich (KISPI) is openly accessible via the Synapse platform (https://www.synapse.org/Synapse:syn23747212/wiki/608434) and will continue to be supported. Training data from other sites is available only under specific agreements, while the testing datasets remain private and are not planned for public release.

2
https://pippiworkshop.github.io/

3 Instructions: https://github.com/fetachallenge/fetachallengesubmission.

4 Available at https://fetachallenge.github.io/pages/Evaluation.

5
https://www.synapse.org/Synapse:syn25649159/wiki/610007

6 Biometry annotation protocol is described on our website: https://fetachallenge.github.io/pages/Data_description

7 **KISPI:** Ethical Committee of the Canton of Zurich, Switzerland (Decision numbers: 2017 00885, 2016 01019, 2017 00167). **CHUV**: Ethics Committee of the Canton de Vaud, Switzerland (CER-VD 2021 00124). **Vienna:** Approved by the ethics review board and data clearing department at the Medical University of Vienna. **UCSF:** institutional review board (IRB 16 20619). **KCL**: Ethics Committee Dulwich (Ethics code 19 LO 0852).

8 Also known as HASTE (Siemens), SSTSE (Philips), or SSFSE (GE), depending on the scanner manufacturer.

9 ↑ means that a higher score is better and ↓ that a lower score is better

10
https://hub.docker.com/r/fetachallenge22/feta-imperial-tum-2022-nnunet

11 The rankings originally published on the website and announced during the MICCAI challenge differ from those presented in this paper due to a change in the ED estimation method. Specifically, we updated the way the ground-truth Euler characteristic is determined. In the original rankings, it was computed based on the manual segmentations. In the current results, it is calculated from manually defined topological properties (see 2.5). Manual segmentations were created via interpolation and because many structures were not segmented on every slice, the resulting ground-truth segmentations contained numerous topological errors (e.g., holes, disconnected components). As a result, they did not reliably represent the expected topological properties of the anatomical structures. To address this, we now use manually specified topological values to calculate the ground-truth EC. We provide additional details on the change in Appendix A11 in the Supplementary materials.
