# Supplementary material for: Advances in automated fetal brain MRI segmentation and biometry: Insights from the FeTA 2024 challenge
Source: Med Image Anal. Author manuscript; Available in PMC 2026 Jul 7. (PMC13338501; doi:10.1016/j.media.2026.103941)
Supplement: 1 [file NIHMS2189672-supplement-1.pdf]

Supplementary Materials

Contents

Appendix A1. Evaluation metrics description 2
Appendix A2. Algorithm descriptions 3
Appendix A3. FeTA 2024 segmentation results by site 73
Appendix A4. FeTA 2024 segmentation results by label 74
Appendix A5. FeTA 2024 segmentation results by pathology 77
Appendix A6. FeTA 2024 biometry results by site, label and pathology 78
Appendix A7. Correlation of quality and challenge metrics 80
Appendix A8. Exploring normalized Dice coefficient 82
Appendix A9. Qualitative examples of predictions 83
Appendix A10. Data acquisition and pre-processing details 88
Appendix A11. Comparison of FeTA 2024 Conference and Paper Rankings 90

Appendix A1. Evaluation metrics description.

Task 1: Segmentation

To evaluate the performance of the segmentation algorithms, we used four complementary metrics:

1. **Dice Similarity Coefficient (Dice)**: Measures voxel-wise correspondence between the predicted and ground truth (GT) segmentations. It is computed as:

$$Dice = \frac{2 \cdot |A \cap B|}{|A| + |B|}$$

where A and B represent the predicted and GT segmentation sets per label, respectively. Higher values of DSC indicate better overlap.

2. **Volume Similarity (VS)**: Assesses similarity of volumes between predicted and GT segmentations, defined as:

$$VS = 1 - \frac{|V_{pred} - V_{GT}|}{|V_{pred} + V_{GT}|}$$

where V_{pred} and V_{GT} are the volumes of the predicted and GT regions, respectively. A value close to 1 indicates high similarity.

3. **Hausdorff Distance (HD95)**: Quantifies contour similarity between predicted and GT segmentations using the 95th-percentile Hausdorff distance:

$$HD95 = \max\left(\max_{x \in A} \min_{y \in B} \|x - y\|, \max_{y \in B} \min_{x \in A} \|x - y\|\right)$$

where A and B are boundary points of the predicted and GT segmentations, and $\| \cdot \|$ denotes the Euclidean distance. Lower HD95 values indicate better contour agreement.

4. **Euler Characteristics (ED) Difference**: Evaluates topological similarity between predicted and GT segmentations, based on Betti numbers. The Euler characteristic is:

$$EC = \text{Betti}_0 - \text{Betti}_1 + \text{Betti}_2$$

where Betti_0 is the number of connected components, Betti_1 the number of loops, and Betti_2 the number of voids. The EC difference is computed as:

$$ED = |EC_{pred} - EC_{GT}|$$

Smaller differences indicate better topological alignment.
The expected ground-truth Betti numbers reflect the typical topology of each fetal brain tissue. Because none of the tissues form loops or enclosed voids in three-dimensional space, all labels have $\text{BN}_1 = 0$ (no 1-dimensional cycles) and $\text{BN}_2 = 0$ (no internal cavities). The 0-dimensional Betti number BN_0 corresponds to the number of connected components. Most tissues—including the eCSF, white matter, ventricular system, cerebellum, deep grey matter, and brainstem—form a single continuous anatomical structure in fetal MRI, and therefore have $\text{BN}_0 = 1$. In contrast, the cortical grey matter is bilaterally distributed across the two cerebral hemispheres and remains separated by the interhemispheric fissure, resulting in two distinct connected components and therefore $\text{BN}_0 = 2$. We provide a summary of selected GT Betti number values for each label in Table 1.

Table 1: A priori ground-truth Betti numbers per tissue label used for ED computation.

**Label**	$\mathbf{BN_0}$	$\mathbf{BN_1}$	$\mathbf{BN_2}$
External CSF (eCSF)	1	0	0
Grey Matter (GM)	2	0	0
White Matter (WM)	1	0	0
Ventricles incl. cavum (VM)	1	0	0
Cerebellum (CBM)	1	0	0
Deep Grey Matter (dGM / SGM)	1	0	0
Brainstem (BSM)	1	0	0

Task 2: Biometry Estimation

The primary metric for evaluating biometry estimation algorithms is **mean average percentage error (MAPE)**, quantifying error relative to actual measurements:

$$MAPE = \frac{1}{N} \sum_{i=1}^{N} \frac{|y_i - \hat{y}_i|}{y_i} \times 100$$

where y_i and $\hat{y}_i$ are the ground truth and predicted measurements, respectively, and N is the total number of measurements.

This metric accounts for variable target structure sizes and assesses the accuracy of the estimated biometric measurements.

Appendix A2. Algorithm descriptions

Team Algorithm Descriptions

The algorithm descriptions are presented for all participating teams in any of the tasks, in the following order:

1. CEMRG
2. falcons
3. feta_sigma
4. hilab
5. Jwcrad
6. lmrcmc
7. LIT
8. mic-dkfz
9. paramahir_2023
10. pasteurdbc
11. unipd-sum-aug
12. UPFetal24
13. vicorob
14. qd_neuroincyte
15. CeSNE-DiGAIR

We summarize in Table 1 the reported hardware specifications, approximate training times, and the number of trainable parameters for the segmentation task, aggregated from the teams' submitted algorithm description documents. This information is limited to what participants explicitly reported: several teams provided joint parameter counts or training times for both tasks (when applicable), and some teams did not report hardware details or parameter counts at all. As a result, the table should be interpreted as a heterogeneous but informative overview rather than a fully standardized comparison.

Table 1: Hardware specification and training time

**Team name**	**Trained on**	**Training time**	**# Parameters**
CEMRG	Unknown	4 hours	Unknown
CeSNE-DiGAIR	NVIDIA GeForce GTX 1080 Ti (12 GB)	75 hours	4,815,745
FALCONS	NVIDIA RTX A5000	26 hours	Unknown
feta_sigma	NVIDIA GeForce RTX 4090	15 hours	106,097,138
hilab	NVIDIA GeForce RTX 2080 Ti	66.7 hours	31,200,424
ichilov-tau-maya	Tesla V100-PCIE-32GB	37 hours	4,701,016
jwcrad	NVIDIA GeForce RTX 4090	35 hours	11,100,000
LIT	3×A100 (80 GB)	90 hours	Unknown
lmrcmc	NVIDIA A800	3 days	140,000,000
mic-dkfz	A100, V100, RTX 2080	16 hours	Unknown
paramahir_2023	RTX 4090 (24 GB VRAM, 30 GB RAM)	10 hours	4,941,726
pasteurdbc	5×NVIDIA A40 (48 GB each)	3 days	61,800,000
qd_neuroincyte	A6000 Ada, RTX 4090	7 days	62,187,002
unipd-sum-aug	NVIDIA A40	10 days	Unknown
UPFetal24	NVIDIA QUADRO RTX 6000	260 hours	Unknown
ViCOROB	3×NVIDIA A30 (24 GB each)	4 days	31,200,000

Appendix A3. FeTA 2024 segmentation results by site

Table A3: Segmentation results for FeTA 2024 by site, presented as mean ± standard deviation.

Team	Site	Dice	HD95	Volume Similarity	Euler diff.
**cemrg_feta**	CHUV	0.819 +- 0.079	2.239 +- 1.641	0.888 +- 0.091	73.271 +- 157.842
	KCL	0.868 +- 0.048	1.482 +- 0.575	0.944 +- 0.050	8.729 +- 17.511
	KISPI	0.775 +- 0.178	3.756 +- 12.339	0.875 +- 0.175	17.150 +- 47.150
	UCSF	0.835 +- 0.064	4.180 +- 10.689	0.941 +- 0.056	21.732 +- 48.451
	VIEN	0.834 +- 0.079	1.846 +- 1.174	0.947 +- 0.056	38.200 +- 76.002
**cesne-digair**	CHUV	0.830 +- 0.060	2.234 +- 1.397	0.927 +- 0.058	29.104 +- 51.562
	KCL	0.856 +- 0.052	1.694 +- 0.524	0.950 +- 0.041	6.257 +- 13.210
	KISPI	0.776 +- 0.154	2.954 +- 2.863	0.890 +- 0.138	9.211 +- 17.841
	UCSF	0.823 +- 0.069	2.126 +- 1.307	0.942 +- 0.050	14.571 +- 25.900
	VIEN	0.814 +- 0.087	2.266 +- 1.687	0.947 +- 0.050	38.132 +- 93.476
**falcons**	CHUV	0.828 +- 0.063	2.054 +- 1.271	0.922 +- 0.056	82.214 +- 166.732
	KCL	0.832 +- 0.061	2.076 +- 1.080	0.906 +- 0.065	17.450 +- 33.388
	KISPI	0.763 +- 0.151	2.890 +- 2.490	0.887 +- 0.139	35.557 +- 80.026
	UCSF	0.430 +- 0.253	21.320 +- 14.077	0.602 +- 0.281	192.339 +- 318.110
	VIEN	0.389 +- 0.275	22.377 +- 17.046	0.580 +- 0.296	134.443 +- 158.795
**feta_sigma**	CHUV	0.823 +- 0.075	2.248 +- 1.659	0.892 +- 0.085	68.571 +- 143.222
	KCL	0.871 +- 0.049	1.469 +- 0.576	0.944 +- 0.051	10.521 +- 23.896
	KISPI	0.772 +- 0.178	3.311 +- 4.349	0.867 +- 0.177	14.814 +- 36.054
	UCSF	0.835 +- 0.064	2.427 +- 3.941	0.937 +- 0.059	21.279 +- 47.773
	VIEN	0.835 +- 0.084	2.216 +- 3.005	0.943 +- 0.062	32.771 +- 65.733
**hilab**	CHUV	0.813 +- 0.079	2.311 +- 1.464	0.878 +- 0.083	66.004 +- 147.774
	KCL	0.851 +- 0.058	1.765 +- 0.823	0.926 +- 0.066	5.807 +- 11.417
	KISPI	0.775 +- 0.176	2.937 +- 3.457	0.878 +- 0.169	13.029 +- 30.945
	UCSF	0.828 +- 0.068	2.677 +- 3.871	0.939 +- 0.053	24.786 +- 62.808
	VIEN	0.831 +- 0.081	2.145 +- 2.654	0.941 +- 0.062	28.832 +- 61.805
**jwcrad**	CHUV	0.741 +- 0.126	4.495 +- 4.241	0.840 +- 0.156	60.668 +- 119.543
	KCL	0.845 +- 0.060	2.101 +- 2.275	0.942 +- 0.059	10.857 +- 25.192
	KISPI	0.780 +- 0.154	3.001 +- 3.122	0.892 +- 0.142	15.054 +- 63.731
	UCSF	0.775 +- 0.129	4.139 +- 12.272	0.908 +- 0.132	22.382 +- 72.242
	VIEN	0.743 +- 0.173	3.375 +- 3.568	0.876 +- 0.174	30.314 +- 48.812
**lit**	CHUV	0.817 +- 0.074	2.397 +- 1.632	0.892 +- 0.078	73.400 +- 150.808
	KCL	0.867 +- 0.049	1.478 +- 0.511	0.950 +- 0.041	7.957 +- 16.179
	KISPI	0.761 +- 0.180	3.400 +- 4.158	0.866 +- 0.176	17.854 +- 49.090
	UCSF	0.812 +- 0.075	2.139 +- 1.852	0.931 +- 0.062	33.982 +- 59.647
	VIEN	0.810 +- 0.093	2.086 +- 2.610	0.937 +- 0.059	51.168 +- 93.347
**lmrcmc**	CHUV	0.814 +- 0.080	2.406 +- 1.409	0.881 +- 0.084	58.054 +- 126.355
	KCL	0.860 +- 0.052	1.611 +- 0.728	0.936 +- 0.058	9.429 +- 21.316
	KISPI	0.774 +- 0.171	3.013 +- 3.697	0.881 +- 0.168	14.675 +- 29.453
	UCSF	0.798 +- 0.084	4.399 +- 6.528	0.933 +- 0.066	38.914 +- 66.956
	VIEN	0.805 +- 0.093	3.682 +- 8.311	0.945 +- 0.057	31.021 +- 50.330
**mic-dkfz-feta24**	CHUV	0.824 +- 0.074	2.125 +- 1.520	0.889 +- 0.083	83.104 +- 190.117
	KCL	0.872 +- 0.046	1.456 +- 0.540	0.950 +- 0.048	8.471 +- 17.095
	KISPI	0.778 +- 0.176	3.667 +- 12.334	0.878 +- 0.175	24.968 +- 98.618
	UCSF	0.849 +- 0.060	1.648 +- 0.929	0.943 +- 0.059	20.679 +- 48.929
	VIEN	0.839 +- 0.077	1.842 +- 1.361	0.947 +- 0.055	34.443 +- 70.139
**paramahir_2023**	CHUV	0.019 +- 0.016	67.984 +- 9.712	0.257 +- 0.191	836.771 +- 514.176
	KCL	0.105 +- 0.114	40.203 +- 38.827	0.665 +- 0.278	1053.286 +- 1194.848
	KISPI	0.036 +- 0.069	134.320 +- 80.816	0.220 +- 0.303	3243.929 +- 2176.151
	UCSF	0.042 +- 0.044	69.115 +- 33.709	0.392 +- 0.246	891.479 +- 1040.067
	VIEN	0.029 +- 0.040	71.887 +- 14.464	0.317 +- 0.263	875.496 +- 803.573
**pasteurdbc**	CHUV	0.816 +- 0.080	2.272 +- 1.574	0.879 +- 0.087	98.582 +- 221.114
	KCL	0.870 +- 0.048	1.494 +- 0.557	0.953 +- 0.043	9.714 +- 19.600
	KISPI	0.770 +- 0.178	3.247 +- 4.094	0.868 +- 0.177	24.864 +- 82.402
	UCSF	0.820 +- 0.076	2.826 +- 3.592	0.920 +- 0.075	21.893 +- 49.927
	VIEN	0.835 +- 0.081	2.040 +- 1.813	0.946 +- 0.060	36.650 +- 71.768
**qd_neuroincyte**	CHUV	0.762 +- 0.127	3.973 +- 3.116	0.877 +- 0.125	27.993 +- 45.480
	KCL	0.800 +- 0.071	15.895 +- 15.467	0.896 +- 0.073	22.821 +- 24.303
	KISPI	0.769 +- 0.174	3.436 +- 4.185	0.878 +- 0.170	19.061 +- 41.108
	UCSF	0.444 +- 0.244	22.082 +- 26.679	0.648 +- 0.288	62.807 +- 133.521
	VIEN	0.689 +- 0.194	9.546 +- 9.233	0.869 +- 0.132	33.057 +- 49.988
**unipd-sum-aug**	CHUV	0.802 +- 0.084	2.517 +- 1.798	0.871 +- 0.093	85.850 +- 191.883
	KCL	0.863 +- 0.047	1.553 +- 0.587	0.950 +- 0.041	13.136 +- 22.792
	KISPI	0.762 +- 0.184	3.169 +- 3.723	0.868 +- 0.180	33.868 +- 108.254
	UCSF	0.827 +- 0.069	2.199 +- 2.283	0.934 +- 0.059	29.396 +- 62.257
	VIEN	0.826 +- 0.077	1.835 +- 0.987	0.945 +- 0.054	54.325 +- 108.289
**upfetal24**	CHUV	0.816 +- 0.080	2.296 +- 1.703	0.882 +- 0.091	89.425 +- 204.043
	KCL	0.844 +- 0.061	2.391 +- 1.490	0.931 +- 0.072	31.729 +- 45.318
	KISPI	0.776 +- 0.175	3.055 +- 3.537	0.876 +- 0.171	19.861 +- 44.504
	UCSF	0.840 +- 0.060	2.452 +- 5.282	0.940 +- 0.056	20.836 +- 51.070
	VIEN	0.837 +- 0.078	1.855 +- 1.405	0.945 +- 0.056	33.868 +- 68.319
**vicorob**	CHUV	0.831 +- 0.070	1.969 +- 1.266	0.899 +- 0.077	93.789 +- 206.532
	KCL	0.869 +- 0.051	1.499 +- 0.659	0.947 +- 0.051	9.986 +- 19.663
	KISPI	0.782 +- 0.170	2.982 +- 3.709	0.881 +- 0.169	19.579 +- 71.933
	UCSF	0.830 +- 0.067	2.276 +- 1.965	0.937 +- 0.056	29.343 +- 68.239
	[illegible]	[illegible]	[illegible]	[illegible]	[illegible]

Appendix A4. FeTA 2024 segmentation results by label

Table A4: Segmentation results for FeTA 2024 by label, presented as mean ± standard deviation.

Team	Label	Dice	HD95	Volume Similarity	Euler diff.
**cemrg_feta**	BS	0.773 ± 0.109	4.066 ± 4.480	0.864 ± 0.128	0.561 ± 0.749
	CBM	0.869 ± 0.121	3.097 ± 15.680	0.933 ± 0.124	0.178 ± 0.718
	CSF	0.805 ± 0.126	3.090 ± 6.242	0.922 ± 0.126	79.172 ± 77.460
	GM	0.748 ± 0.075	1.885 ± 4.882	0.921 ± 0.062	144.156 ± 183.114
	SGM	0.801 ± 0.109	3.417 ± 4.473	0.863 ± 0.124	1.056 ± 1.166
	VM	0.864 ± 0.061	2.028 ± 6.517	0.951 ± 0.046	2.389 ± 2.444
	WM	0.892 ± 0.038	2.269 ± 5.881	0.961 ± 0.032	13.161 ± 14.716
**cesne-digair**	BS	0.790 ± 0.091	3.665 ± 2.187	0.899 ± 0.091	0.206 ± 0.525
	CBM	0.868 ± 0.088	1.466 ± 0.716	0.945 ± 0.083	0.367 ± 1.624
	CSF	0.794 ± 0.108	2.726 ± 2.860	0.937 ± 0.080	49.006 ± 35.876
	GM	0.730 ± 0.077	1.557 ± 0.786	0.935 ± 0.043	87.644 ± 109.380
	SGM	0.798 ± 0.105	3.061 ± 1.881	0.879 ± 0.125	0.833 ± 0.647
	VM	0.848 ± 0.066	1.713 ± 1.228	0.946 ± 0.045	1.578 ± 1.468
	WM	0.883 ± 0.043	2.030 ± 0.835	0.964 ± 0.030	6.817 ± 9.445
**falcons**	BS	0.519 ± 0.310	11.287 ± 10.191	0.688 ± 0.288	28.778 ± 41.150
	CBM	0.570 ± 0.361	19.103 ± 22.806	0.721 ± 0.288	42.172 ± 94.277
	CSF	0.608 ± 0.244	12.110 ± 11.915	0.719 ± 0.265	175.044 ± 316.827
	GM	0.604 ± 0.177	10.279 ± 11.330	0.845 ± 0.145	289.128 ± 272.968
	SGM	0.542 ± 0.324	9.231 ± 16.580	0.617 ± 0.334	17.350 ± 25.692
	VM	0.762 ± 0.158	5.269 ± 5.874	0.863 ± 0.144	44.506 ± 74.144
	WM	0.791 ± 0.145	10.000 ± 11.203	0.903 ± 0.116	108.122 ± 164.206
**feta_sigma**	BS	0.776 ± 0.104	4.263 ± 4.436	0.865 ± 0.118	0.483 ± 0.751
	CBM	0.867 ± 0.124	2.042 ± 3.452	0.925 ± 0.128	0.244 ± 0.759
	CSF	0.802 ± 0.132	3.058 ± 5.381	0.911 ± 0.136	83.772 ± 73.078
	GM	0.750 ± 0.076	1.438 ± 0.766	0.922 ± 0.063	121.089 ± 165.583
	SGM	0.800 ± 0.112	3.196 ± 2.267	0.860 ± 0.128	1.083 ± 1.143
	VM	0.869 ± 0.057	1.293 ± 0.696	0.951 ± 0.040	2.261 ± 2.175
	WM	0.893 ± 0.038	1.722 ± 0.536	0.962 ± 0.032	13.039 ± 12.755
**hilab**	BS	0.772 ± 0.098	4.155 ± 3.437	0.874 ± 0.108	0.350 ± 0.523
	CBM	0.866 ± 0.121	1.931 ± 3.170	0.924 ± 0.123	0.067 ± 0.310
	CSF	0.802 ± 0.121	2.506 ± 3.554	0.916 ± 0.125	79.889 ± 89.962
	GM	0.740 ± 0.086	1.492 ± 0.743	0.917 ± 0.068	114.972 ± 170.136
	SGM	0.790 ± 0.107	3.513 ± 3.317	0.860 ± 0.124	0.750 ± 0.838
	VM	0.853 ± 0.071	1.672 ± 2.266	0.931 ± 0.067	2.439 ± 2.077
	WM	0.889 ± 0.039	1.768 ± 0.550	0.955 ± 0.039	12.394 ± 13.574
**jwcrad**	BS	0.676 ± 0.175	5.253 ± 3.670	0.779 ± 0.212	7.417 ± 74.544
	CBM	0.799 ± 0.153	3.189 ± 3.409	0.878 ± 0.164	2.989 ± 9.602
	CSF	0.774 ± 0.121	2.693 ± 3.000	0.926 ± 0.093	62.778 ± 55.944
	GM	0.705 ± 0.088	1.931 ± 1.834	0.908 ± 0.085	105.406 ± 138.418
	SGM	0.737 ± 0.155	6.690 ± 15.354	0.827 ± 0.176	9.600 ± 74.649
	VM	0.829 ± 0.098	2.808 ± 3.244	0.931 ± 0.088	4.700 ± 6.119
	WM	0.866 ± 0.081	2.420 ± 2.004	0.950 ± 0.064	15.317 ± 16.491
**lit**	BS	0.770 ± 0.103	4.159 ± 2.815	0.872 ± 0.119	0.511 ± 0.639
	CBM	0.866 ± 0.120	1.787 ± 3.629	0.935 ± 0.118	0.083 ± 0.394
	CSF	0.782 ± 0.131	2.615 ± 3.683	0.917 ± 0.123	78.561 ± 69.369
	GM	0.718 ± 0.081	1.652 ± 1.190	0.916 ± 0.067	184.161 ± 166.182

	SGM	0.793 ± 0.116	3.250 ± 2.395	0.862 ± 0.136	0.933 ± 1.122
	VM	0.848 ± 0.063	1.487 ± 1.074	0.924 ± 0.060	2.361 ± 2.044
	WM	0.878 ± 0.054	1.790 ± 0.867	0.951 ± 0.036	13.983 ± 21.532
**lmrcmc**	BS	0.761 ± 0.112	4.559 ± 5.464	0.871 ± 0.116	1.333 ± 2.422
	CBM	0.855 ± 0.108	3.499 ± 7.590	0.927 ± 0.111	3.850 ± 11.014
	CSF	0.799 ± 0.120	2.689 ± 5.592	0.932 ± 0.115	52.617 ± 48.115
	GM	0.713 ± 0.076	2.053 ± 2.993	0.910 ± 0.077	124.883 ± 138.935
	SGM	0.782 ± 0.116	4.384 ± 3.510	0.852 ± 0.132	3.722 ± 8.246
	VM	0.846 ± 0.077	2.597 ± 6.345	0.945 ± 0.053	5.006 ± 7.943
	WM	0.876 ± 0.044	2.474 ± 4.290	0.953 ± 0.036	37.844 ± 72.378
**mic-dkfz-feta24**	BS	0.792 ± 0.102	3.430 ± 2.265	0.877 ± 0.116	6.250 ± 74.563
	CBM	0.875 ± 0.121	2.452 ± 14.827	0.936 ± 0.124	5.589 ± 74.609
	CSF	0.811 ± 0.123	2.492 ± 3.960	0.921 ± 0.124	76.667 ± 79.789
	GM	0.754 ± 0.078	1.342 ± 0.664	0.919 ± 0.068	153.933 ± 222.976
	SGM	0.803 ± 0.112	2.981 ± 1.915	0.859 ± 0.128	1.278 ± 1.303
	VM	0.867 ± 0.060	1.275 ± 0.655	0.953 ± 0.046	2.144 ± 2.387
	WM	0.895 ± 0.036	1.598 ± 0.464	0.961 ± 0.032	14.583 ± 15.262
**paramahir_2023**	BS	0.002 ± 0.004	80.114 ± 52.805	0.439 ± 0.300	1315.700 ± 1572.594
	CBM	0.003 ± 0.011	98.476 ± 46.129	0.471 ± 0.319	1182.661 ± 1613.381
	CSF	0.057 ± 0.068	75.774 ± 55.679	0.364 ± 0.283	1668.756 ± 1510.524
	GM	0.040 ± 0.038	77.447 ± 54.473	0.284 ± 0.275	1239.539 ± 1637.509
	SGM	0.034 ± 0.039	81.118 ± 51.993	0.238 ± 0.230	1424.194 ± 1523.328
	VM	0.049 ± 0.056	74.136 ± 54.399	0.328 ± 0.273	2102.550 ± 1503.443
	WM	0.092 ± 0.105	78.237 ± 53.946	0.238 ± 0.238	982.206 ± 1700.717
**pasteurdbc**	BS	0.771 ± 0.110	4.197 ± 2.852	0.863 ± 0.124	5.939 ± 74.584
	CBM	0.867 ± 0.109	1.809 ± 1.441	0.925 ± 0.112	0.217 ± 1.135
	CSF	0.804 ± 0.132	2.797 ± 4.533	0.918 ± 0.136	75.700 ± 75.225
	GM	0.735 ± 0.082	1.517 ± 0.790	0.909 ± 0.079	184.989 ± 256.428
	SGM	0.795 ± 0.116	3.224 ± 2.146	0.850 ± 0.132	1.072 ± 1.237
	VM	0.862 ± 0.064	1.333 ± 0.902	0.945 ± 0.055	2.056 ± 1.902
	WM	0.884 ± 0.050	2.439 ± 3.815	0.951 ± 0.048	20.678 ± 21.555
**qd_neuroincyte**	BS	0.564 ± 0.322	16.358 ± 33.573	0.679 ± 0.343	31.933 ± 164.379
	CBM	0.699 ± 0.287	12.927 ± 18.047	0.813 ± 0.238	6.317 ± 11.709
	CSF	0.642 ± 0.164	7.771 ± 4.291	0.826 ± 0.140	50.983 ± 42.466
	GM	0.604 ± 0.156	6.012 ± 5.081	0.882 ± 0.104	85.433 ± 61.912
	SGM	0.693 ± 0.171	10.169 ± 9.490	0.791 ± 0.174	13.939 ± 25.437
	VM	0.765 ± 0.161	11.839 ± 11.728	0.894 ± 0.104	26.578 ± 37.313
	WM	0.797 ± 0.120	8.013 ± 6.939	0.902 ± 0.106	24.883 ± 26.451
**unipd-sum-aug**	BS	0.762 ± 0.119	4.033 ± 2.908	0.857 ± 0.133	6.178 ± 74.569
	CBM	0.857 ± 0.102	2.000 ± 1.808	0.929 ± 0.107	0.278 ± 0.702
	CSF	0.798 ± 0.138	2.282 ± 3.628	0.917 ± 0.139	85.600 ± 90.364
	GM	0.726 ± 0.086	1.499 ± 0.758	0.910 ± 0.078	215.856 ± 226.509
	SGM	0.794 ± 0.111	3.363 ± 2.449	0.863 ± 0.132	1.222 ± 1.194
	VM	0.854 ± 0.067	1.443 ± 0.958	0.937 ± 0.055	2.400 ± 2.590
	WM	0.885 ± 0.038	1.708 ± 0.486	0.954 ± 0.038	15.144 ± 16.221
**upfetal24**	BS	0.780 ± 0.113	4.028 ± 2.620	0.871 ± 0.132	1.611 ± 3.007
	CBM	0.872 ± 0.108	1.529 ± 1.116	0.935 ± 0.109	0.350 ± 1.339
	CSF	0.810 ± 0.121	2.556 ± 4.235	0.921 ± 0.122	84.133 ± 80.372
	GM	0.742 ± 0.077	1.401 ± 0.708	0.917 ± 0.074	164.483 ± 233.666
	SGM	0.790 ± 0.108	3.652 ± 4.141	0.850 ± 0.128	1.611 ± 3.941

	VM	0.861 ± 0.064	1.636 ± 3.494	0.942 ± 0.051	3.061 ± 4.046
	WM	0.889 ± 0.035	2.082 ± 3.293	0.954 ± 0.036	24.522 ± 35.431
**vicorob**	BS	0.788 ± 0.104	3.744 ± 2.639	0.886 ± 0.109	6.372 ± 74.563
	CBM	0.873 ± 0.101	1.616 ± 1.192	0.932 ± 0.106	0.167 ± 0.780
	CSF	0.807 ± 0.124	2.370 ± 3.839	0.919 ± 0.127	97.094 ± 91.240
	GM	0.745 ± 0.076	1.415 ± 0.774	0.919 ± 0.070	169.622 ± 238.677
	SGM	0.801 ± 0.112	3.079 ± 2.062	0.868 ± 0.131	0.928 ± 1.041
	VM	0.868 ± 0.059	1.405 ± 1.703	0.956 ± 0.042	2.706 ± 2.315
	WM	0.891 ± 0.039	1.677 ± 0.504	0.957 ± 0.035	12.161 ± 12.048

Appendix A5. FeTA 2024 segmentation results by pathology

Table A5: Segmentation results for FeTA 2024 by pathology, presented as mean ± standard deviation.

**Team**	**Pathology**	**Dice**	**HD95**	**Volume Similarity**	**Euler diff.**
**cemrg_feta**	Neurotypical	0.838 ± 0.078	1.949 ± 1.685	0.923 ± 0.083	36.990 ± 104.012
	Pathological	0.808 ± 0.127	3.596 ± 10.477	0.910 ± 0.122	32.150 ± 79.136
**cesne-digair**	Neurotypical	0.834 ± 0.072	2.037 ± 1.401	0.939 ± 0.063	17.255 ± 36.003
	Pathological	0.801 ± 0.114	2.557 ± 2.138	0.921 ± 0.095	24.059 ± 65.344
**falcons**	Neurotypical	0.695 ± 0.233	9.431 ± 15.061	0.819 ± 0.212	89.800 ± 155.566
	Pathological	0.571 ± 0.297	12.417 ± 13.435	0.720 ± 0.285	110.080 ± 229.547
**feta_sigma**	Neurotypical	0.838 ± 0.079	1.944 ± 1.509	0.922 ± 0.082	35.382 ± 95.466
	Pathological	0.809 ± 0.128	2.846 ± 4.160	0.907 ± 0.125	28.568 ± 69.351
**hilab**	Neurotypical	0.832 ± 0.079	2.029 ± 1.465	0.917 ± 0.080	33.253 ± 97.439
	Pathological	0.802 ± 0.126	2.781 ± 3.629	0.906 ± 0.120	27.445 ± 72.466
**jwcrad**	Neurotypical	0.796 ± 0.110	3.051 ± 3.409	0.901 ± 0.121	33.539 ± 88.625
	Pathological	0.746 ± 0.163	4.013 ± 8.390	0.872 ± 0.166	26.496 ± 68.179
**lit**	Neurotypical	0.830 ± 0.080	1.971 ± 1.471	0.922 ± 0.079	40.859 ± 102.670
	Pathological	0.789 ± 0.132	2.751 ± 3.317	0.902 ± 0.122	39.423 ± 85.891
**lmrcmc**	Neurotypical	0.822 ± 0.086	2.884 ± 5.872	0.921 ± 0.082	31.589 ± 78.737
	Pathological	0.790 ± 0.126	3.432 ± 4.936	0.906 ± 0.118	33.745 ± 71.625
**mic-dkfz-feta24**	Neurotypical	0.842 ± 0.076	1.844 ± 1.368	0.925 ± 0.080	41.797 ± 130.019
	Pathological	0.817 ± 0.126	2.550 ± 8.005	0.912 ± 0.121	33.278 ± 93.104
**paramahir_2023**	Neurotypical	0.041 ± 0.066	75.381 ± 49.828	0.365 ± 0.293	1316.442 ± 1491.284
	Pathological	0.038 ± 0.060	85.358 ± 55.674	0.314 ± 0.282	1502.144 ± 1709.552
**pasteurdbc**	Neurotypical	0.834 ± 0.082	2.000 ± 1.522	0.918 ± 0.085	46.558 ± 146.162
	Pathological	0.802 ± 0.129	2.879 ± 3.591	0.901 ± 0.126	37.212 ± 98.278
**qd_neuroincyte**	Neurotypical	0.744 ± 0.178	9.321 ± 15.672	0.867 ± 0.156	31.234 ± 71.161
	Pathological	0.626 ± 0.242	11.399 ± 16.616	0.792 ± 0.233	36.915 ± 78.492
**unipd-sum-aug**	Neurotypical	0.827 ± 0.083	2.041 ± 1.614	0.917 ± 0.087	49.062 ± 132.939
	Pathological	0.797 ± 0.131	2.582 ± 2.797	0.903 ± 0.125	44.620 ± 111.600
**upfetal24**	Neurotypical	0.832 ± 0.080	2.096 ± 1.579	0.918 ± 0.087	45.657 ± 131.983
	Pathological	0.811 ± 0.124	2.682 ± 4.136	0.909 ± 0.120	35.099 ± 88.130
**vicorob**	Neurotypical	0.841 ± 0.077	1.870 ± 1.600	0.929 ± 0.076	46.210 ± 138.108
	Pathological	0.811 ± 0.122	2.457 ± 2.685	0.911 ± 0.117	37.085 ± 97.032

Appendix A6. FeTA 2024 biometry results by site, label and pathology

In this section of the supplementary materials, we present detailed results for the second task of the FeTA 2024 challenge, which focuses on fetal brain biometry estimation. To complement the primary evaluation metrics, we also report the **Mean Absolute Error (MAE)**, which offers an intuitive interpretation of the prediction errors in physical units (millimeters). We also further analyze our results relative to the [GA] baseline to better understand where the proposed methods bring improvement.

MAE analysis. The MAE is defined as:

$$\mathrm{MAE} = \frac{1}{N}\sum_{i=1}^{N} |\hat{y}_i - y_i|$$

where N is the number of samples, $\hat{y}_i$ is the predicted measurement for the i-th subject, and y_i is the corresponding ground truth measurement.

All MAE values are reported in millimeters (mm), which facilitates a direct understanding of the magnitude of the errors made by the algorithms in the context of fetal brain structure dimensions.

Table A6.1. Biometry results for all teams participating in the FeTA 2024 biometry challenge together with the baseline model and inter-rater variability stratified by label. The values for each team are sorted by MAPE in the increasing order.

Team	Pathology	MAE (mm)	MAPE
`[GA]`	Pathological	3.468±4.504	0.090±0.120
	Neurotypical	2.948±3.057	0.103±0.137
`[inter-rater]`	Neurotypical	1.333±1.781	0.041±0.057
	Pathological	1.625±2.040	0.065±0.105
**cesne-digair**	Neurotypical	3.283±6.208	0.092±0.126
	Pathological	2.896±4.831	0.099±0.139
**falcons**	Neurotypical	10.262±14.883	0.262±0.281
	Pathological	13.525±14.425	0.411±0.421
**feta_sigma**	Neurotypical	2.447±2.567	0.069±0.074
	Pathological	3.499±4.260	0.123±0.168
**jwcrad**	Neurotypical	1.948±1.779	0.057±0.057
	Pathological	3.123±6.939	0.095±0.143
**paramahir_2023**	Pathological	9.564±11.359	0.257±0.235
	Neurotypical	13.438±15.592	0.306±0.262
**pasteurdbc**	Neurotypical	3.704±3.551	0.139±0.177
	Pathological	3.852±3.698	0.176±0.254
**qd_neuroincyte**	Pathological	14.208±19.129	0.384±0.452
	Neurotypical	19.497±26.977	0.419±0.433

Table A6.2. Biometry results for all teams participating in the FeTA 2024 biometry challenge together with the baseline model and inter-rater variability stratified by label

Team	Team	MAE (mm)	MAPE
[GA]	HV	1.409±1.388	0.113±0.131
	LCC	3.406±3.302	0.127±0.161
	TCD	2.787±3.415	0.108±0.167
	bBIP	4.156±4.864	0.068±0.074
	sBIP	4.363±4.691	0.065±0.064
[inter-rater]	HV	0.993±0.885	0.080±0.088
	LCC	2.542±3.541	0.096±0.142
	TCD	1.189±1.233	0.049±0.072
	bBIP	1.852±1.388	0.033±0.028
	sBIP	0.951±0.950	0.015±0.016
cesne-digair	HV	1.197±1.199	0.098±0.120
	LCC	5.704±3.329	0.177±0.102
	TCD	3.238±3.914	0.123±0.162
	bBIP	2.381±3.250	0.040±0.055
	sBIP	3.079±10.121	0.047±0.147
falcons	HV	5.747±4.488	0.463±0.507
	LCC	9.804±8.722	0.349±0.349
	TCD	10.294±10.779	0.367±0.342
	bBIP	14.906±17.945	0.246±0.274
	sBIP	18.980±20.905	0.281±0.293
feta_sigma	HV	1.429±1.273	0.116±0.121
	LCC	3.540±3.073	0.126±0.145
	TCD	3.213±3.489	0.137±0.203
	bBIP	3.369±3.453	0.057±0.060
	sBIP	3.507±5.144	0.055±0.075
jwcrad	HV	1.377±1.087	0.103±0.083
	LCC	3.241±3.047	0.112±0.117
	TCD	1.983±1.735	0.072±0.072
	bBIP	3.285±7.850	0.054±0.131
	sBIP	3.013±7.579	0.048±0.132
paramahir_2023	HV	4.148±3.911	0.294±0.243
	LCC	8.936±8.877	0.285±0.244
	TCD	9.459±9.225	0.308±0.270
	bBIP	16.397±16.698	0.261±0.242
	sBIP	17.728±18.415	0.255±0.244
pasteurdbc	HV	5.694±2.572	0.435±0.262
	LCC	5.833±5.816	0.205±0.230
	TCD	1.261±1.381	0.054±0.099
	bBIP	3.820±2.663	0.065±0.046
	sBIP	2.470±1.637	0.037±0.027
qd_neuroincyte	HV	5.762±5.638	0.428±0.528
	LCC	9.946±10.714	0.328±0.379
	TCD	15.352±15.694	0.479±0.413
	bBIP	24.821±29.450	0.384±0.432
	sBIP	26.958±32.977	0.378±0.436

Table A6.3. Biometry results for all teams participating in the FeTA 2024 biometry challenge together with the baseline model and inter-rater variability stratifies by site

Team	Site	MAE (mm)	MAPE
[GA]	KCL	2.365±2.297	0.055±0.045
	CHUV	3.027±2.790	0.083±0.121
	UCSF	3.280±4.375	0.094±0.121
	VIEN	3.788±5.361	0.106±0.112
	KISPI	3.262±3.219	0.121±0.172
[inter-rater]	KCL	0.747±0.645	0.020±0.024
	CHUV	1.019±0.924	0.029±0.029
	UCSF	1.421±1.513	0.050±0.059
	KISPI	1.753±1.814	0.069±0.095
	VIEN	2.168±3.070	0.086±0.135
cesne-digair	UCSF	2.487±3.767	0.079±0.094
	KCL	3.109±3.544	0.085±0.116
	KISPI	2.610±4.410	0.096±0.147
	VIEN	3.665±9.285	0.106±0.157
	CHUV	3.551±3.629	0.109±0.133
falcons	KCL	4.885±6.207	0.109±0.108
	CHUV	11.379±22.126	0.262±0.427
	KISPI	7.156±5.066	0.282±0.311
	VIEN	14.906±13.615	0.435±0.457
	UCSF	18.260±12.581	0.508±0.230
feta_sigma	KCL	1.779±1.603	0.046±0.047
	CHUV	2.164±2.126	0.069±0.116
	KISPI	2.567±2.734	0.111±0.189
	VIEN	3.540±3.598	0.117±0.122
	UCSF	4.402±5.357	0.121±0.119
jwcrad	KCL	1.305±1.025	0.035±0.035
	CHUV	2.392±1.896	0.071±0.079
	UCSF	2.499±5.267	0.074±0.096
	KISPI	2.047±1.704	0.078±0.085
	VIEN	4.051±9.407	0.108±0.182
paramahir_2023	KCL	4.153±4.023	0.107±0.105
	KISPI	4.483±3.553	0.161±0.184
	VIEN	7.354±6.228	0.177±0.102
	UCSF	6.523±5.287	0.185±0.137
	CHUV	30.396±16.278	0.677±0.044
pasteurdbc	KCL	3.515±2.607	0.119±0.139
	CHUV	3.129±2.686	0.127±0.175
	UCSF	3.663±3.287	0.167±0.224
	KISPI	3.749±2.977	0.179±0.283
	VIEN	4.774±5.341	0.182±0.221
qd_neuroincyte	KCL	5.122±8.736	0.094±0.132
	KISPI	3.080±3.803	0.122±0.208
	VIEN	7.525±10.180	0.234±0.461
	UCSF	15.855±13.145	0.429±0.262
	CHUV	45.380±30.413	0.958±0.296

`[GA]` baseline analysis. Breaking down the results across neurotypical and pathological groups, we see on Table A6.4 that although MAPE errors are slightly higher for pathological subjects, the ordering does not drastically change: teams that outperform the `[GA]` baseline do so in both settings. This is further confirmed when breaking down results across sites and structures, as highlighted in Figure 1: generally, the team `jwcrad` outperforms consistently the `[GA]` baseline, with exceptions in the LCC and HV breakdown for neurotypical subjects, where the performance is difficult to distinguish between the two models.

Table A6.4. Metrics and ranking for the biometry estimation task broken down by neurotypical and pathological subjects, sorted according to the pathological MAPE. [GA] and [inter-rater] entries do not represent participating models, thus their rank is marked as *

	Neurotypical		Pathological	
Team	MAPE	Rank	MAPE	Rank
`[inter-rater]`	$0.041_{\pm 0.057}$	*	$0.065_{\pm 0.105}$	*
jwcrad	$0.057_{\pm 0.057}$	1	$0.095_{\pm 0.143}$	1
cesne-digair	$0.092_{\pm 0.126}$	3	$0.099_{\pm 0.139}$	2
`[GA]`	$0.070_{\pm 0.080}$	*	$0.118_{\pm 0.156}$	*
feta_sigma	$0.069_{\pm 0.074}$	2	$0.123_{\pm 0.168}$	3
pasteurdbc	$0.139_{\pm 0.177}$	4	$0.176_{\pm 0.254}$	4
paramahir_2023	$0.306_{\pm 0.262}$	6	$0.257_{\pm 0.235}$	5
qd_neuroincyte	$0.419_{\pm 0.433}$	7	$0.384_{\pm 0.452}$	6
falcons	$0.262_{\pm 0.281}$	5	$0.411_{\pm 0.421}$	7

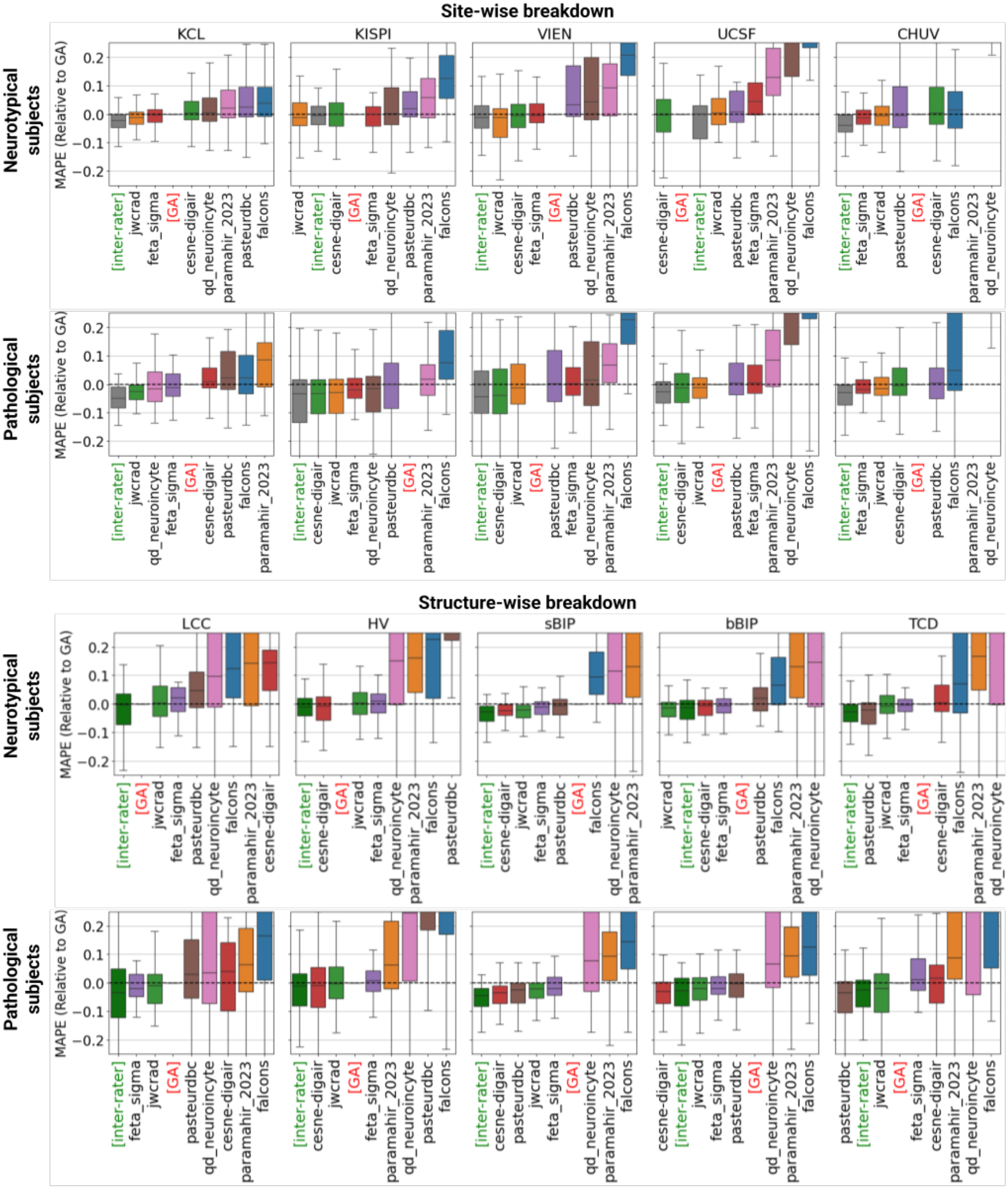

Figure 1: Biometry MAPE relative to the `[GA]` baselines across sites (top) and labels (bottom) for all teams participating in the Task 2 together with the GA baseline model ([GA]) and the inter-rater variability [inter-rater]. Each plot is broken down by neurotypical and pathological subjects. Teams are sorted in ascending order for each subplot independently, based on their mean MAPE for a given site or label.

Appendix A7. Correlation of quality and challenge metrics

Correlation plots of visual quality scores and metrics across different sites and super-resolution methods for the best teams in FeTA 2024 Each dot represents an average metric value(of the top 3 teams in FeTA2024 `cesne-digair, mic-dkfz-feta24, vicorob`) for a given subject, with blue indicating quality scores (left axis) and red indicating a given metric (right axis). Sites and methods are grouped on the x-axis. Dashed lines connect data points for individual subjects across metrics. Pearson correlation coefficients (r) between quality and Dice are shown above each group

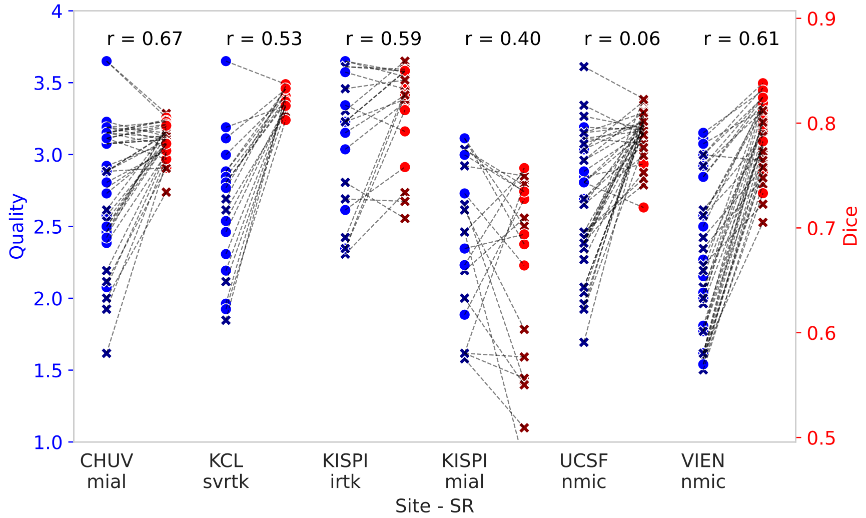

Figure A7.1: Correlation between visual quality scores and Dice across different sites and super-resolution methods.

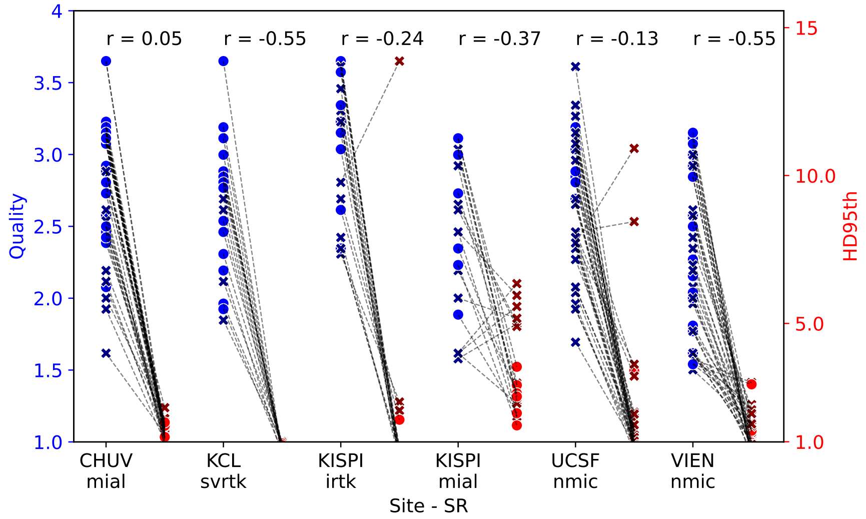

Figure A7.2: Correlation between visual quality scores and HD95 across different sites and super-resolution methods.

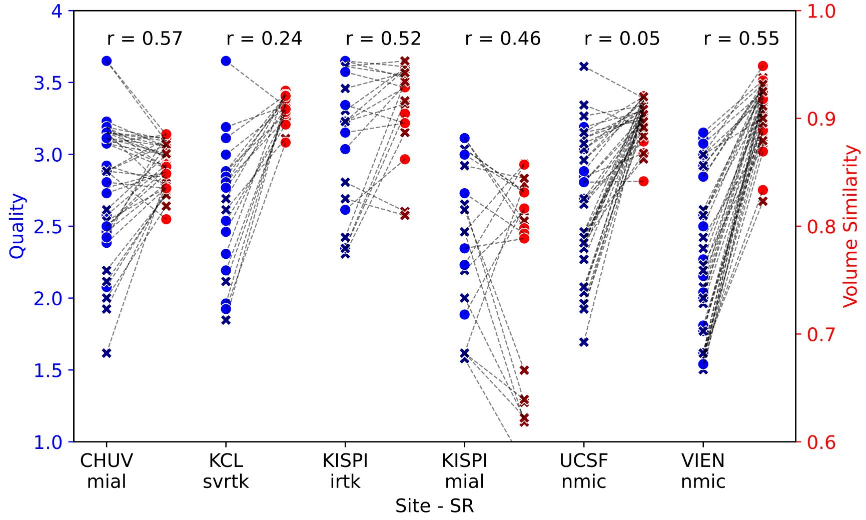

Figure A7.3: Correlation between visual quality scores and VS across different sites and super-resolution methods.

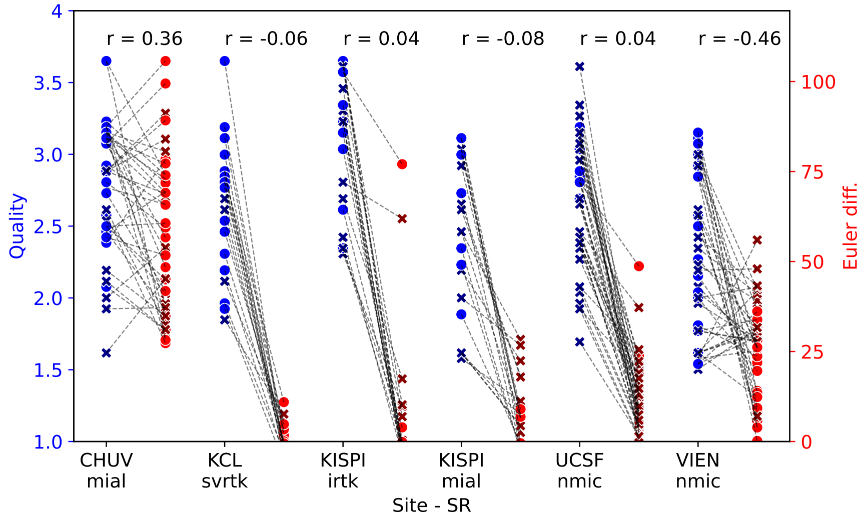

Figure A7.4: Correlation between visual quality scores and ED across different sites and super-resolution methods.

Appendix A8. Exploring normalized Dice coefficient

Correlation between Dice, Normalized Dice, Volume, and GA In Figure A8.1 (a), we present the distribution of Pearson correlation coefficients between each team's Dice score and the volume of the segmented label, averaged across all subjects for the 15 participating teams in the FeTA 2024 challenge. We observe that using the normalized Dice coefficient mitigates the known bias of the standard Dice metric toward larger volumes. However, normalized Dice does not eliminate the correlation with gestational age (GA). This residual association may be due to GA acting as a complex confounding factor, potentially interacting with other variables such as acquisition site and pathology, rather than directly influencing the metric.

Impact of Using Normalized Dice on Team Rankings In Figure A8.1 (b), we assess the stability of team rankings when using normalized Dice instead of the standard Dice score. Although normalized Dice reduces the volume-related bias, the correlation structure across teams appears relatively stable. Rankings remain largely consistent, with only two pairs of teams swapping positions. Notably, the top six teams maintain their original ranking when switching from Dice to normalized Dice, suggesting that the relative performance differences are robust to this normalization.

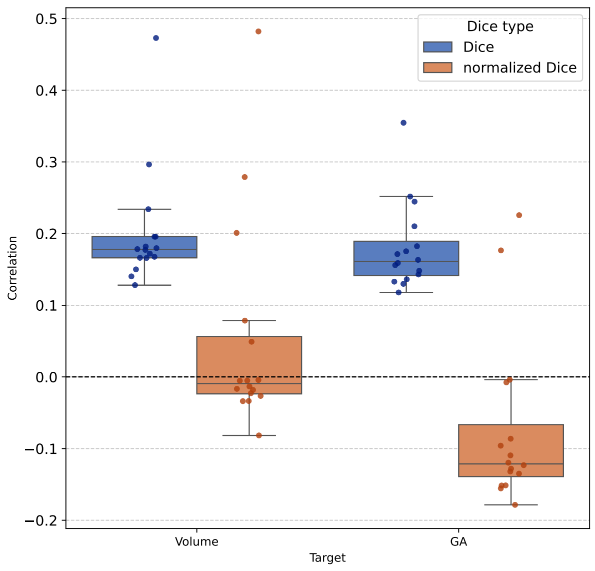

(a) Correlation coefficients between Dice/normalized Dice with volume and GA.

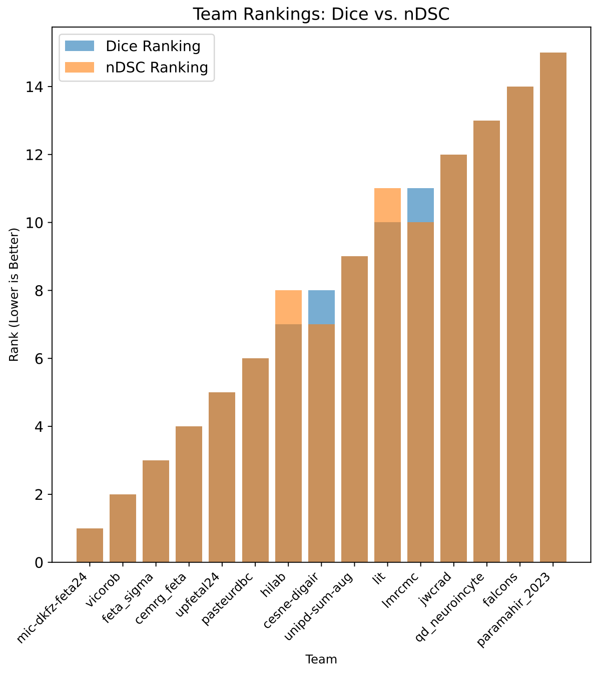

(b) Team-ranking stability using Dice vs. normalized Dice.

Figure A8.1: (a) Per-team Dice vs. volume/GA correlations. (b) Impact of normalization on team rankings.

Appendix A9. Qualitative examples of predictions

Qualitative Results This section of the supplementary materials presents qualitative comparisons of the predictions made by the top four teams in the FeTA 2024 challenge.

Figure A9.1 shows representative segmentation outputs for five subjects from the testing set, while Figure A9.2 highlights the corresponding segmentation errors. Overall, the visual differences between the top-performing models are minimal, indicating that all four algorithms deliver highly consistent and accurate segmentations in well-defined cases.

Figures A9.3 and A9.4 illustrate cases with the lowest Dice scores across the evaluated models. These difficult cases are typically associated with poor image quality (e.g., Subjects 2 and 3), or signs of abnormal fetal brain development (e.g., Subjects 0, 1, and 4). Such challenges underscore the limitations of current methods when dealing with low-quality inputs or atypical anatomy.

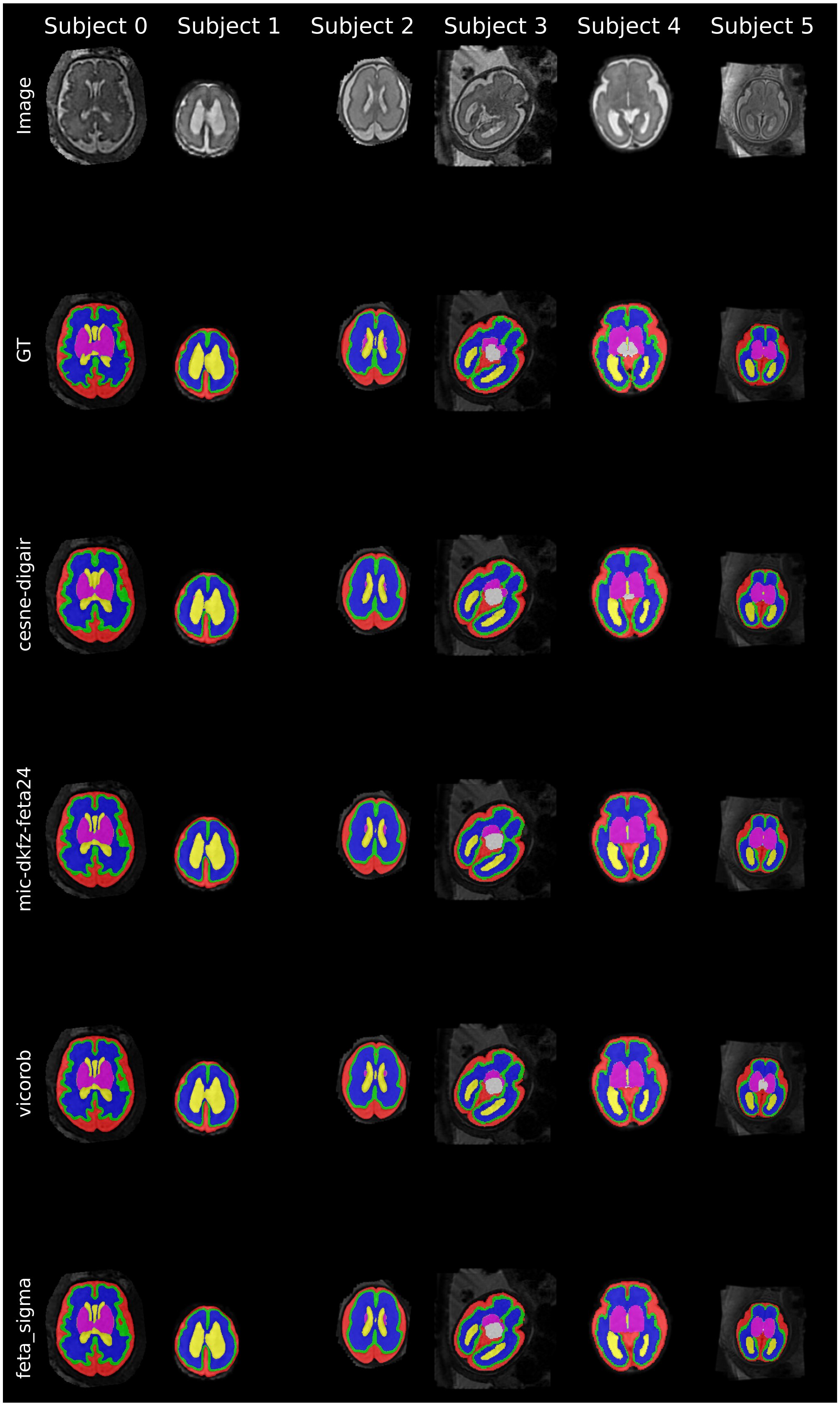

Figure A9.1: Segmentation results for five testing subjects produced by the top four teams in the FeTA 2024 challenge.

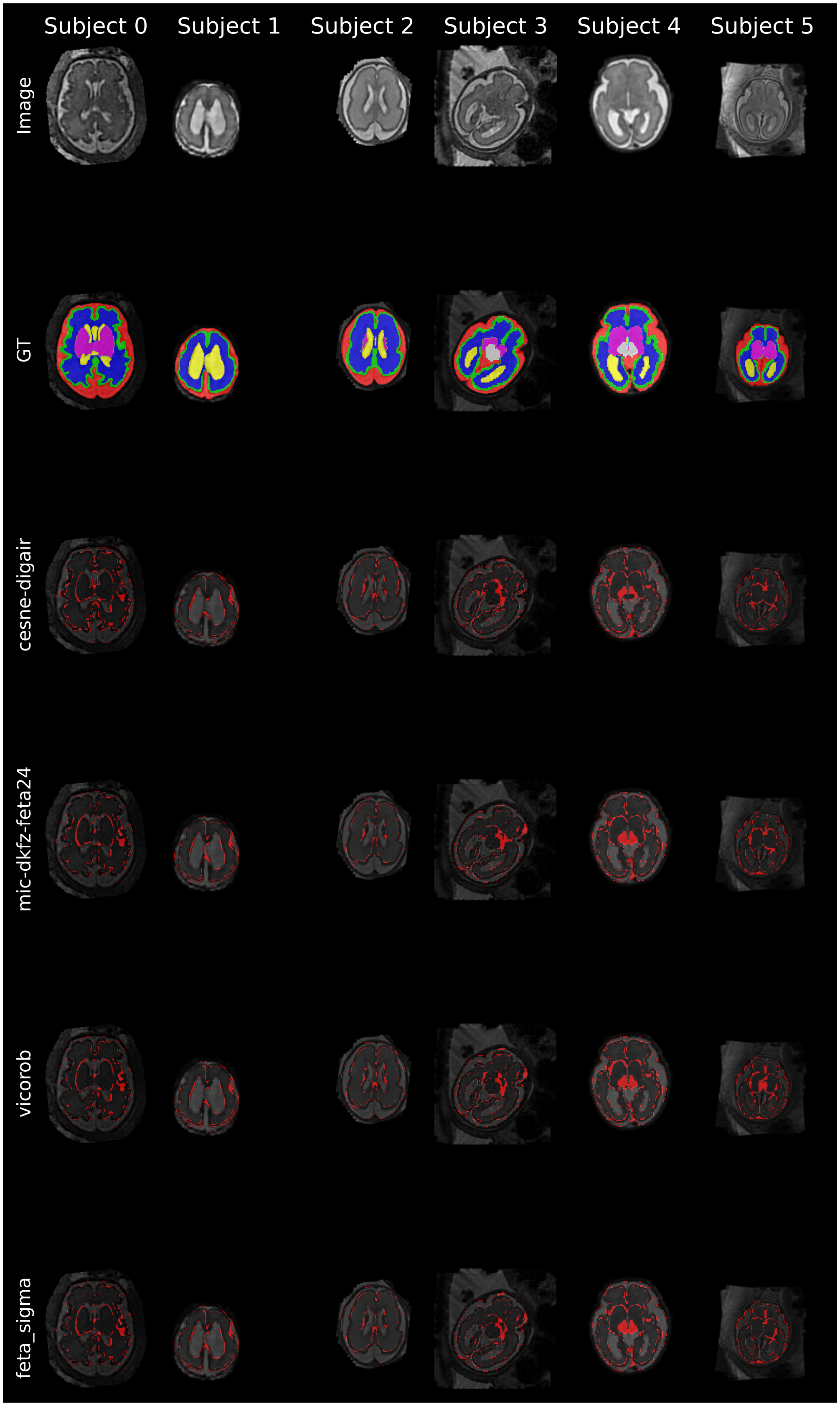

Figure A9.2: Segmentation errors for five testing subjects produced by the top four teams in the FeTA 2024 challenge. Red regions indicate voxels where predicted labels differ from the ground truth.

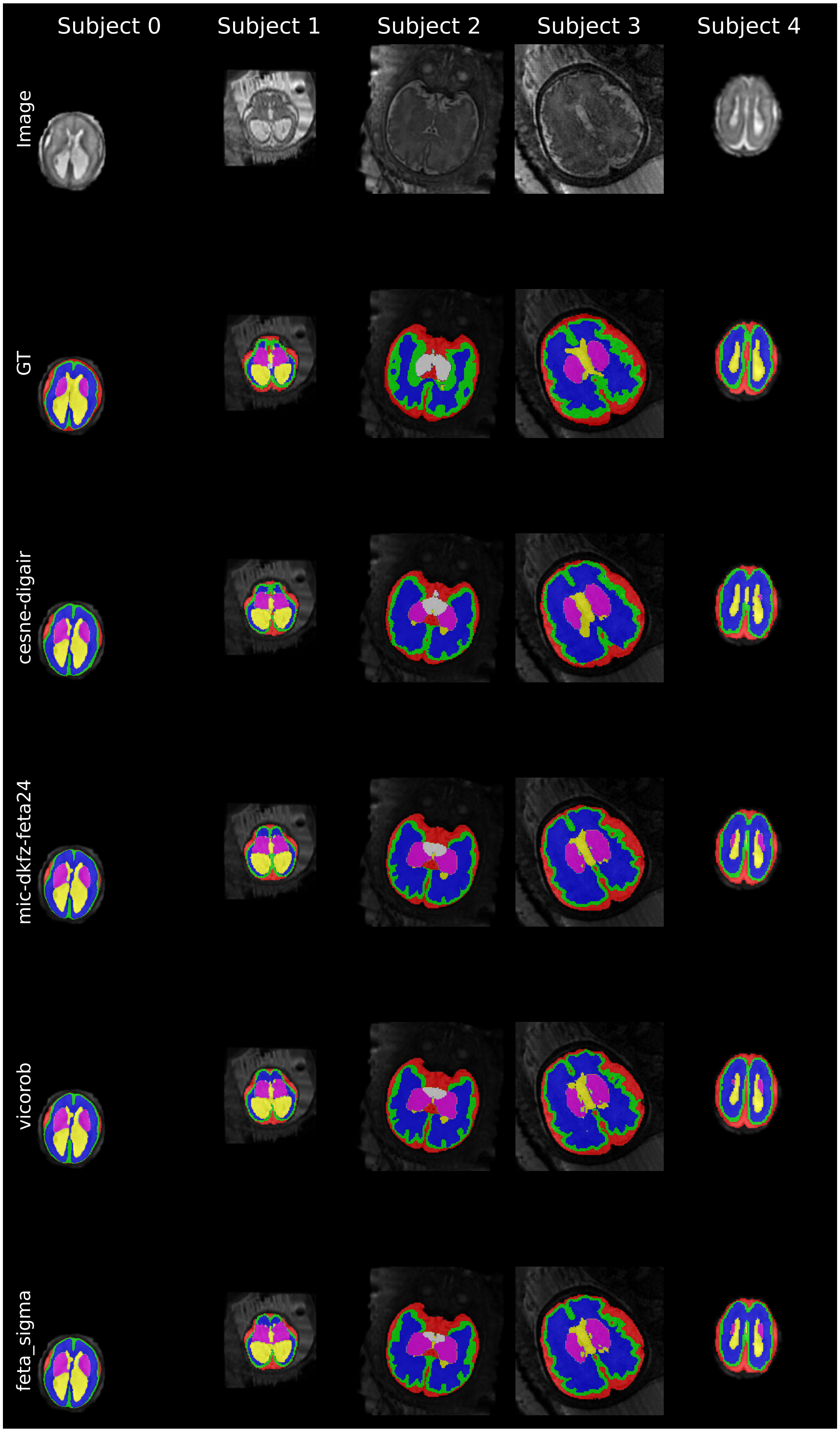

Figure A9.3: Segmentation results for five challenging testing subjects with the lowest Dice scores, produced by the top four teams in the FeTA 2024 challenge.

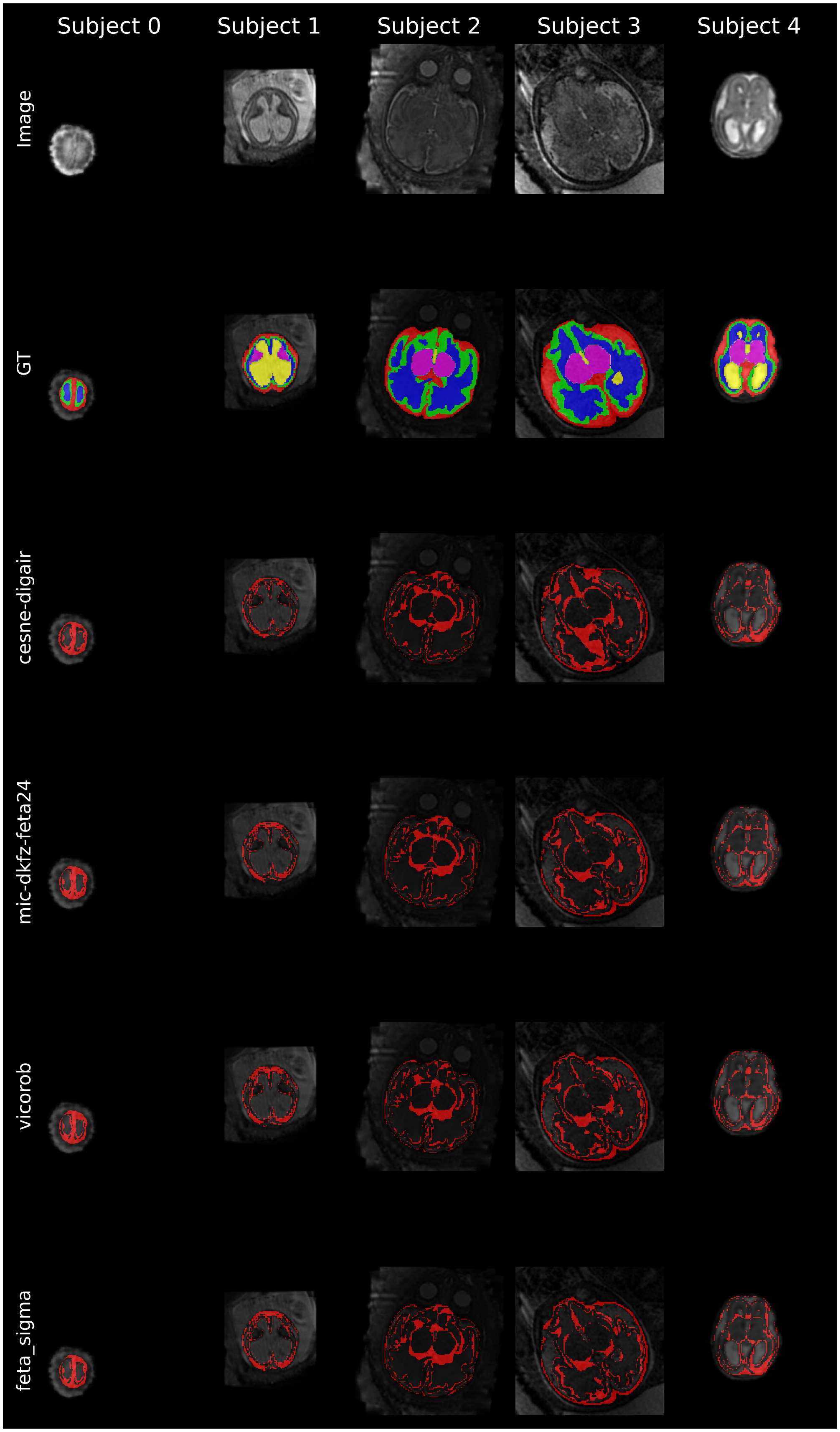

Figure A9.4: Segmentation errors for the five challenging testing subjects shown in Figure A9.3. Red voxels indicate mismatches between the predicted labels and ground truth.

Appendix A10. Data acquisition and pre-processing details

The datasets used in the FeTA 2024 Challenge were curated from five different institutions, combining both previously released and newly collected data. They were selected to span a broad range of gestational ages and to include both neurotypical and pathological cases, thereby reflecting real-world clinical diversity. Each case consisted of a 3D super-resolution reconstruction (SRR) of the fetal brain, accompanied, in the training set, by manually annotated label maps for seven tissue classes and five biometric measurements. The information provided here is identical to that available in the official challenge data description [1], which accompanied the challenge proposal on Zenodo.

KISPI Dataset

The Kispi dataset was acquired at the University Children's Hospital Zurich (Switzerland). Mothers provided general or specific research consent for the reuse of fetal MRI data. Ethical approval was granted by the Ethics Committee of the Canton of Zurich (decision numbers 2017_00885, 2016_01019, 2017_00167), and a waiver was obtained for releasing a fully anonymised dataset. Imaging was performed on clinical 1.5T and 3T GE Signa Discovery MR450 and MR750 scanners, using either an 8-channel cardiac coil or a body coil, without maternal or fetal sedation. T2-weighted single-shot fast spin-echo (SSFSE) sequences were acquired with in-plane resolution of $0.5\times0.5\,\mathrm{mm}^2$ and slice thickness of 3–5 mm, with TR values between 2000–3500 ms, TE of 120 ms (minimum), and a 90° flip angle. Field of view (200–240 mm) and matrix size (1.5T: 256×224; 3T: 320×224) were adjusted depending on GA and fetal size. Images were oriented relative to the fetal brain, with axial, coronal, and sagittal stacks acquired. SRR was performed using either the MIALSRTK or IRTK-simple pipeline, yielding an isotropic resolution of $0.5\,\mathrm{mm}^3$.

CHUV Dataset

The CHUV dataset was collected at the Lausanne University Hospital (Switzerland). Mothers were scanned as part of routine clinical care, and data were retrospectively gathered from January 2013 to April 2021. All images were anonymised, and ethical approval for reuse was obtained from the Canton de Vaud Ethics Committee (CERVD 2021_00124), including approval for release of an anonymised dataset for non-medical reproducible research. Imaging was performed on a 1.5T Siemens MAGNETOM Aera scanner, without maternal or fetal sedation, using an 18-channel body coil and a 32-channel spine coil. T2-weighted HASTE sequences were acquired in three orthogonal orientations, with at least two acquisitions per orientation. Acquisition parameters included TR/TE of 1200/90 ms, flip angle of 90°, echo train length of 224, echo spacing of 4.08 ms, field of view of $360\times360\,\mathrm{mm}^2$, voxel size of $1.13\times1.13\times3.00\,\mathrm{mm}^3$, and a 10% inter-slice gap, with acquisition times between 26–36 s. SRR was performed using the MIALSRTK pipeline to achieve $1.125\,\mathrm{mm}^3$ isotropic resolution.

Vienna Dataset

The Vienna dataset was acquired at the General Hospital Vienna / Medical University of Vienna (Austria) as part of a retrospective single-centre study, approved by the local ethics review board and data clearing department. Imaging was performed on 1.5T Philips Ingenia/Intera and 3T Philips Achieva systems, using a cardiac coil and without maternal or fetal sedation. For each case, at least three T2-weighted ssFSE sequences (TE 80–140 ms) were acquired in three orthogonal planes (axial, coronal, sagittal) relative to the fetal brainstem axis and/or corpus callosum axis. Slice thickness was between 3–5 mm with a gap of 0.3–1 mm, pixel spacing ranged from 0.65–1.17 mm, and acquisition time ranged from 13.46 to 41.19 s. SRR was performed using NiftyMIC, producing $0.5\,\mathrm{mm}^3$ isotropic resolution volumes.

UCSF Dataset

The UCSF dataset was acquired at the University of California, San Francisco (USA). Clinical fetal MRI scans were approved for anonymised retrospective research use (IRB 16_20619), and the ethical approval was extended via a data sharing agreement to permit external anonymised data sharing. Imaging was performed on 3T GE Discovery MR750 or MR750W (wide-bore) scanners with a 32-channel cardiac coil, without

maternal or fetal sedation. For each subject, at least three T2-weighted ssFSE sequences were acquired, one per orientation (sagittal, axial, coronal), with a 240 mm field of view, a 512×512 matrix, yielding an in-plane resolution of approximately $0.5 \times 0.5\,\text{mm}^2$ and 3 mm slice thickness. TR ranged from 2000–3500 ms, TE exceeded 100 ms, and flip angle was 90°. SRR was performed using NiftyMIC, achieving $0.8\,\text{mm}^3$ isotropic resolution.

KCL Dataset

The KCL dataset was acquired at St Thomas' Hospital, London (UK) as part of a prospective single-centre study, fully anonymised in line with local procedures. Ethical approval was granted by the Ethics Committee Dulwich (19_LO_0852). Imaging was performed on a 0.55T Siemens MAGNETOM Free.Max system, using the Contour L coil and integrated spine coil, with the mother in a supine position and without sedation. T2-weighted HASTE sequences were acquired in three orthogonal orientations, typically covering the brain in all three views plus three additional whole-uterus stacks. Acquisition parameters included TR/TE of 2500/106 ms, flip angle of 180°, field of view of $450 \times 450\,\text{mm}^2$, base resolution of 304×304, voxel size of $1.5 \times 1.5 \times 4.5\,\text{mm}^3$, and acquisition times between 64–122 s. SRR was performed using SVRTK to produce volumes with $0.8\,\text{mm}^3$ isotropic resolution.

References

[1] Bach Cuadra, Meritxell and Payette, Kelly and Jakab, Andras and Licandro, Roxane and Barkovich, Matt and Li, Hongwei Bran and Roulet, Margaux and Zalevskyi, Vladyslav and Sanchez, Thomas and Fajardo Rojas, Diego and Li, Liu and Hutter, Jana, Fetal Tissue Annotation Challenge. *Zenodo*, April 2024. doi:10.5281/zenodo.10986046.

Appendix A11. Comparison of FeTA 2024 Conference and Paper Rankings

ED Evaluation Change

During the preparation of this manuscript, we undertook an extended methodological review of the topology-based evaluation used in the challenge. This led to a refinement in how the Euler characteristic difference (ED) was computed for the segmentation ranking (while all other metrics remained unchanged).

For the conference leaderboard, the ground-truth Euler characteristic and the corresponding Betti numbers were derived directly from the manual segmentations. These reference masks were produced in a single anatomical plane, and, as is common in such settings, the resulting 3D volumes contained numerous small topological inconsistencies that are difficult to correct manually. Consequently, the empirically derived Betti numbers showed substantial variability and deviated considerably from anatomical expectations for several structures. Table 1 summarizes the mean and standard deviation of these empirical Betti numbers and the resulting Euler characteristic across tissues.

Table 1. Mean+-std of topology related metrics for the manually derived GT.

Label	$Betti_0$ (mean±std)	$Betti_1$	$Betti_2$	Euler characteristic
eCSF	36.81±57.46	128.58±109.31	1.20±2.17	-6.91±26.22
GM	13.02±26.04	181.11±246.11	0.85±2.09	-15.53±49.97
WM	17.28±26.42	23.38±22.90	0.23±0.59	-2.51±10.46
VM	2.96±1.67	3.39±2.81	0.18±0.61	-1.51±5.17
CBM	1.14±0.80	0.08±0.43	0.02±0.15	0.16±0.55
SGM	1.20±0.55	0.87±1.09	0.03±0.18	0.47±2.58
BS	28.81±372.60	28.29±372.64	28.31±372.64	0.04±0.47

Given these observations and the prior anatomical knowledge detailed in Appendix A1, we adopted a more principled approach for the manuscript: we specified *a priori*, anatomically plausible Betti numbers for each tissue class and used these fixed values to compute the ground-truth Euler characteristic for ED, similarly to how it was done in previous challenge editions. This ensured a stable, interpretable, and semantically meaningful topology-based comparison across methods.

This refinement resulted in a shift in the final segmentation rankings compared to the conference website. For full transparency, Table 2 reports both sets of rankings side-by-side. Differences arise solely from the updated ED computation and from the exclusion of one team ("ichilov-tau-maya"), who declined participation in the manuscript.

Table 2. Task 1 (Segmentation): comparison of final rankings. ΔRank = Website − Paper.

**Team**	**Website rank**	**Paper rank**	**ΔRank**
mic-dkfz-feta24	1	2	-1
cemrg_feta	2	5	-3
vicorob	2	3	-1
feta_sigma	4	4	0
pasteurdbc	5	11	-6
upfetal24	6	6	0
lit	7	8	-1
cesne-digair	8	1	+7
hilab	9	7	+2
unipd-sum-aug	10	10	0
lmrcmc	11	9	+2
jwcrad	12	11	+1
qd_neuroincyte	13	13	0
falcons	14	14	0
ichilov-tau-maya	15	–	–
paramahir_2023	16	15	+1
